# Application of Positive Psychology in Digital Interventions for Children, Adolescents, and Young Adults: Systematic Review and Meta-Analysis of Controlled Trials

**DOI:** 10.2196/56045

**Published:** 2024-08-14

**Authors:** Sundas Saboor, Adrian Medina, Laura Marciano

**Affiliations:** 1 Department of Health Behavior & Health Education University of Michigan School of Public Health Ann Arbor, MI United States; 2 Deptartment of Social & Behavioral Sciences Harvard T.H. Chan School of Public Health Boston, MA United States; 3 Lee Kum Sheung Center for Health and Happiness Department of Social and Behavioral Sciences Harvard T.H. Chan School of Public Health Boston, MA United States

**Keywords:** positive psychology, digital interventions, ill-being, well-being, systematic review, meta-analysis, smartphone, mobile phone

## Abstract

**Background:**

The rising prevalence of mental health issues in children, adolescents, and young adults has become an escalating public health issue, impacting approximately 10%-20% of young people on a global scale. Positive psychology interventions (PPIs) can act as powerful mental health promotion tools to reach wide-ranging audiences that might otherwise be challenging to access. This increased access would enable prevention of mental disorders and promotion of widespread well-being by enhancing self-efficacy, thereby supporting the achievement of tangible objectives.

**Objective:**

We aimed to conduct a comprehensive synthesis of all randomized controlled trials and controlled trials involving children, adolescents, and young adults, encompassing both clinical and nonclinical populations, to comprehensively evaluate the effectiveness of digital PPIs in this age group.

**Methods:**

After a literature search in 9 electronic databases until January 12, 2023, and gray literature until April 2023, we carried out a systematic review of 35 articles, of which 18 (51%) provided data for the meta-analysis. We included randomized controlled trials and controlled trials mainly based on web-based, digital, or smartphone-based interventions using a positive psychology framework as the main component. Studies included participants with a mean age of <35 years. Outcomes of PPIs were classified into indicators of well-being (compassion, life satisfaction, optimism, happiness, resilience, emotion regulation and emotion awareness, hope, mindfulness, purpose, quality of life, gratitude, empathy, forgiveness, motivation, and kindness) and ill-being (depression, anxiety, stress, loneliness, and burnout). PRISMA (Preferred Reporting Items for Systematic Reviews and Meta-Analyses) guidelines were used for the selection of studies and data extraction. Quality assessment was performed following the CONSORT (Consolidated Standards of Reporting Trials) guidelines.

**Results:**

For well-being outcomes, meta-analytic results showed that PPIs augmented the feeling of purpose, gratitude, and hope (Hedges *g*=0.555), compassion (Hedges *g*=0.447), positive coping behaviors (Hedges *g*=0.421), body image–related outcomes (Hedges *g*=0.238), and positive mindset predisposition (Hedges *g*=0.304). For ill-being outcomes, PPIs reduced cognitive biases (Hedges *g*=–0.637), negative emotions and mood (Hedges *g*=–0.369), and stress levels (Hedges *g*=–0.342). Of note, larger effect sizes were found when a waiting list control group was considered versus a digital control group. A funnel plot showed no publication bias. Meta-regression analyses showed that PPIs tended to show a larger effect size on well-being outcomes in studies including young adults, whereas no specific effect was found for ill-being outcomes.

**Conclusions:**

Revised evidence suggests that PPIs benefit young people’s well-being and mitigate ill-being symptoms. Digital platforms offer a unique way to address their mental health challenges, although not without limitations. Future research should explore how they work for the needs of the young population and further examine what specific PPIs or combination of interventions is most beneficial with respect to other digital control groups.

**Trial Registration:**

PROSPERO International Prospective Register of Systematic Reviews CRD42023420092; https://www.crd.york.ac.uk/prospero/display_record.php?RecordID=420092

## Introduction

### Background

Mental health problems among children, adolescents, and young adults are a growing public health concern, affecting 10% to 20% of young people worldwide [1]. According to World Mental Health Report 2022, 970 million people have mental disorders, with 3% to 7% of mental disorders among those aged <10 years, 13.5% to 14.7% among those aged 10 to 19 years, and 14.1% to 14.9% among those aged 20 to 49 years [2]. Globally, 1 in 7 (14%) people who are aged 10 to 19 years have different mental health conditions, and most of them remain untreated [3]. In addition, among three-quarters of adults, long-term mental health problems occurred before the age of 24 years [1]. According to the US Surgeon General’s Advisory Report, from 2009 to 2019, the proportion of high school students with persistent feelings of sadness or hopelessness increased by 40% [4]. In addition, the mental health conditions of young people faced unprecedented challenges during the COVID-19 pandemic, wherein the risk of depression and anxiety doubled [4], together with feelings of loneliness [5,6].

Considering the above statistics, enhancing youth well-being is an urgent public health need and concern today [7]. To date, the field of psychiatry and psychology have primarily addressed challenges in treating mental illness, focusing on therapy access and engagement [8] only after a symptomatology has occurred. However, there has been less attention on enhancing and protecting mental well-being [8,9] before the onset of mental health issues. Interventions aiming at diminishing mental health problems by using the prevailing disease model of human functioning (ie, ill-being model) largely ignore positive psychological assets such as meaning, courage, compassion, and kindness that could not only relieve ill-being states but also prevent them [10]. Positive psychology aims to provide a more comprehensive scientific knowledge of the human experience, from positive to negative, and better integrate and complement the illness framework with concepts related to positive mental health and use them at scale to solve public health issues [10].

Advocating for a more holistic approach to mental health promotion and expanding the field’s focus to include strategies for improving mental well-being are crucial to boost intervention effectiveness, prevent mental illness and relapse, and broaden our understanding of how to support individuals in flourishing and improving their overall quality of life [9]. Tomé et al [11] conducted a systematic review on 13 studies, focusing on children and adolescents aged 0 to 18 years who had been a target audience for mental health and well-being promotion interventions and suggested that preventive school-based interventions can reduce the onset and progression of clinical disorders and promote good mental health. Another systematic review and meta-analysis of 16 studies concluded that people with severe mental illness benefit from positive psychology interventions (PPIs) in terms of enhancing mental health [12]. Williams et al [13] proposed in their systematic review that social interventions to increase positive emotions for people diagnosed with mental health disorders are suitable and effective adjuncts to mental health treatment.

Hence, in this systematic review and meta-analysis, we aimed to focus on digital interventions based on positive psychology as a promising option to promote well-being and prevent mental health issues in a population (children, adolescents, and young adults) that is not at a high risk of developing such issues.

### Positive Psychology Framework

According to the American Psychological Association, *positive psychology* is defined as “a field of psychological theory and research that focuses on the psychological states (e.g., contentment, joy), individual traits or character strengths (e.g., intimacy, integrity, altruism, wisdom), and social institutions that enhance subjective well-being and make life most worth living” [14]. It is the scientific study of optimal functioning that identifies skills and strengths so that an individual or a community can thrive [15]. Positive psychology complements and extends the ill-being framework: PPIs focus on the science of positive mental states and behavioral patterns to improve quality of life [16,17]. Positive psychology involves the promotion of well-being differentiated between hedonic well-being, focusing on happiness, pleasure attainment, and pain avoidance, and eudaimonic well-being, related to meaning, self-realization, and full functioning of the person [18]. By doing so, positive psychology solves problems by identifying and leveraging individual and societal strengths [19]. Also, positive psychology enhances the importance of flourishing, a construct that encompasses positive emotions and relationships, engagement, meaning, and accomplishments directly or indirectly related to different dimensions of well-being, including psychological, emotional, social, and subjective [20].

Martin EP Seligman, the father of positive psychology, introduced 5 dimensions essential for well-being known as the PERMA model: positive emotions, engagement, relationships, meaning, and accomplishment [21,22]. Positive emotions (eg, joy, interest, contentment, and love) serve as markers of flourishing and optimal well-being [23]. Engagement is the extent of use and a subjective experience characterized by interest, affect, and attention [24]. Positive relationships can be regarded as strong connections with family and friends, developing a sense of belonging [25]. Meaning is understood as coherence, purpose, and a sense of life’s inherent value, making it worth living [26]. Accomplishment refers to achievement, mastery, and competence [27].

### Digital PPIs

On the basis of positive psychology theories and empirical research, PPIs aim to ameliorate well-being and health outcomes by increasing positive feelings, healthier lifestyle behaviors, and better cognitive functioning [28]. PPIs promote positive well-being outcomes, especially in people dealing with stress, by fostering positive daily emotions, providing people with stress-free time, mindful attention and positive cognition, and strengthening social relationships based on the Positive Pathways to Health theoretical model [29-31]. This theoretical model posits that PPIs promote physical and psychological well-being for people dealing with stress by elevating positive emotions experienced in their daily lives [30]. PPIs rely on elements such as optimism, spirituality, hopefulness, happiness, gratitude, creativity, meaning, and purpose [32].

From a public health perspective, PPIs can serve as effective mental health promotion tools to reach large target audiences, which would be challenging to reach otherwise. PPIs can be used as preventive and easily accessible tools that can promote well-being at scale by building self-efficacy and reinforcing the effects of meeting concrete goals [33]. Health promotion strategies can address complex mental health issues, treat preclinical and underdiagnosed cases, and prevent the recurrence of health problems to sustain health networks [34]. These strategies bolster public policies such as providing employment opportunities and antidiscriminatory laws, establishing supportive environments through interventions such as parenting programs, strengthening community action through initiatives such as media campaigns and research, and improving health services such as depression screening, all aimed at enhancing health and well-being [10].

Although mental health problems are a growing public health concern among youth, research on the impact of digital PPIs on this population remains limited. Prior reviews primarily addressed conventional interventions, such as in-person therapies within clinical settings and nonclinical settings [35-38]. However, considering the ongoing digitalization of health care, web-based resources and mental health applications have emerged as new avenues for young individuals to access health care services. Surprisingly, there is a notable absence of previous reviews exclusively focusing on digital PPIs for this demographic.

In previous reviews, individual meta-analyses for interventions were assessed for behavioral interventions [39] and ecological momentary interventions [40]. Other meta-analyses were performed for well-being components individually, for example, optimism [41], anxiety [42,43], depression [44-48], well-being [46,47,49], employee or work-based well-being [50-52], happiness [49], and school-based well-being [53]. However, previous reviews and meta-analyses excluded studies that did not mention outcome measure of well-being [54]; had no restriction on age groups [47,55]; or included only adults [56,57] or clinical population (eg, cardiovascular disease, psychiatric or somatic disorder, medical patients, schizophrenia, severe mental illness, and chronic pain) [12,42,58-61]. Although these reviews provide in-depth analysis of the effects of PPIs, the effects of digital PPIs on children, adolescents, and young adults have not been consistently summarized. Moreover, previous reviews mainly included traditional interventions (eg, cognitive therapy or cognitive behavioral therapy [CBT], mindfulness CBT, face-to-face group therapies and meditation, mainly among college students, young community members, or pediatric clinical settings) [41,47,62]; however, with the digitalization process, web-based resources and mental health apps are becoming the new way for youth to access health services. However, no previous reviews focused only on digital PPIs. Finally, when it comes to included studies, few reviews and meta-analyses included design such as randomized controlled trials (RCTs) and controlled trials (CTs) [41,47,62].

### Study Aim

To overcome the abovementioned limitations, our objective was to comprehensively synthesize all RCTs and CTs conducted with young population (ie, children, adolescents, and young adults), encompassing both clinical and nonclinical populations, in order to assess the global effectiveness of digital PPIs on individuals in this age group holistically, without differentiation of prevention and treatment. In particular, we aimed to carry out a systematic review and meta-analysis including both clinical and nonclinical population to determine the efficacy of digital PPIs by considering if digital PPIs maintain health (by improving well-being constructs of compassion, life satisfaction, optimism, happiness, hope, resilience, etc or by reducing ill-being constructs of depression, anxiety, stress, loneliness, burnout, etc) and if there is any difference with respect to other (digital) control conditions.

In this study, we described study characteristics, theoretical background of the PPIs, quality assessment of the studies, the diverse range of PPIs used by the studies, the well-being and ill-being outcomes of these PPIs, and the meta-analytic results for the outcomes.

## Methods

### Overview

This systematic review and meta-analysis followed the PRISMA (Preferred Reporting Items for Systematic Reviews and Meta-Analyses) guidelines [63].

### Study Sources and Searches

In total, 9 electronic databases (Communication & Mass Media Complete, Psychology and Behavioral Sciences Collection, PsycINFO, CINAHL, ERIC: Education Resource Information Center, MEDLINE Proquest, ProQuest Sociology, Web of Science [ISI Web of Knowledge], and MEDLINE PubMed) were searched up to January 12, 2023 (Textbox 1).

All citations were imported into Zotero reference manager (Corporation for Digital Scholarship) to automatically remove any duplicates. An additional hand search was carried out by scanning the references of relevant review articles identified along with all gray literature in Google Scholar until April 2023.

**Keywords used in the search string combined by the Boolean operator “AND.”**

**Keywords**

- Online* OR internet* OR digital* OR smartphone* OR social media OR EMI* OR EMA* OR in-situ OR ecological momentary assess* OR ecological momentary intervention* OR ESM* OR experience sampling* OR ambulatory assess* OR trace data OR chatbot* OR artificial intelligence* OR AI* OR conversational agent* OR chatterbot* OR virtual agent*
- positive psychology* OR positive psychotherapy* OR kindness* OR optimism* OR gratitude* OR happ* OR flourish* OR satisfaction* OR optimis* OR strength* OR forgiveness* OR positive relationship* OR savoring* OR altruism* OR gift* OR meaning* OR purpose* OR hedon* OR eudaimon* OR compassion* OR hop*
- Interven* OR treatment* OR therap* OR RCT* OR random* OR trial* OR control*

### Study Selection

After duplicates were removed from the initial list of extracted publications, 2 authors independently completed title and abstract screening. For title and abstract screening, we included studies that (1) were either RCTs or CTs; (2) had the intervention that was mainly based on positive psychology (eg, gratitude, hope, optimism, etc) as the main component (for interventions, we included psychotherapies, therapy, interventions, mindfulness, training, exercise, and similar); and (3) included children, adolescents, and young adults with a mean age of <35 years. Although mental health disorders and treatment vary among this diverse age range, aggregating mental health across this range is appropriate due to similarities in mental health challenges and responses to interventions observed across different developmental stages within this age range. The proportion of individuals with the onset of any mental disorders before the ages of 14, 18, and 25 years were 34.6%, 48.4%, and 62.5%, respectively, and the peak age was 14.5 years [64]. Separation anxiety disorder, specific phobia, and social phobia have their mean onset before the age of 15 years, whereas agoraphobia, obsessive-compulsive disorder, posttraumatic stress disorder, panic disorder, and generalized anxiety disorder began, on average, between 21.1 and 34.9 years [65]. The mean age of <35 years is in line with the upper age limit of the early psychosis paradigm reporting on universal interventions or selective interventions [66-68]. In addition, studies needed to (4) include from both clinical and nonclinical population and (5) be carried out web-based or digital or through smartphone-based interventions. We excluded studies with face-to-face interventions and psychotherapy only of any kind, including digital or web-based or smartphone based. We also excluded studies in which positive psychology was not the main focus of the intervention (eg, when positive psychology was an additional component of a mindfulness-based intervention or CBT or other therapies). Moreover, we removed studies with no experimental design or control group and studies where the average age of the sample was >35 years or the focus was on caregivers. We further excluded conference abstracts, theses, books, or book sections. We excluded studies that were not in the English language. If at least 1 of the 2 authors decided to retain an article during the title and abstract screening process, we included it in the full-text screening. Discrepancies after full-text screening were solved through a consensus meeting with a third author.

### Data Extraction

For each included study, we extracted information about the article (first author, year of publication, journal, and title); the study (country where the study was conducted, study design, sample size of experimental group, presence of control group, type of control group, sample size of control group, type of sampling, and attrition rates); and characteristics of the sample (including clinical or general population with details, ethnicity, gender distribution, and age). For intervention, we extracted information regarding the kind of positive intervention and its details, reference theory of the intervention, the type of activity and intervention, the setting of the intervention with details, the duration of the intervention, number of follow-ups and the time of the follow-ups, and data collection survey details. Outcomes included different ill-being and well-being constructs. Finally, we collected information on intervention evaluation, statistical analyses, and results to be converted into effect sizes.

### Quality Assessment

In total, 2 authors independently assessed the quality of the studies according to the CONSORT (Consolidated Standards of Reporting Trials) guidelines [69], and a sum score was created, with a higher score indicating methodological quality. CONSORT guidelines are better suited for assessing the quality of study reporting for RCTs and CTs as recommended by Altman [70] and Versluis et al [40]. As a form of quality assessment, we checked whether studies explicitly mentioned (1) title and abstract; (2) introduction (including background and objectives); (3) methods (including trial design, participants, interventions, outcomes, sample size, randomization-sequence generation, randomization-sequence allocation concealment, randomization-implementation, blinding, and statistical methods); (4) results (including participants flow diagram, participant flow, recruitment, baseline data, number analyzed, outcomes and estimations, ancillary analyses, and harms); (5) discussion (including limitations, interpretations, generalizability, and registration); and (6) other information such as funding and protocol. For each paper, we rated if each criterion of the quality assessment was 0=“absent” and 1=“completely met.” Then, we calculated a sum score of all criteria. The maximum score obtainable for each study was 34.

### Data Synthesis and Analysis

We conducted the meta-analysis using “meta” [27] packages in R statistical software (R Foundation for Statistical Computing). A standardized mean difference approach was used as a measure of effect size using the Hedges adjusted *g*, which is similar to Cohen *d*, but it includes an adjustment for small sample bias [71]. All the analyses were implemented using the inverse-variance method with a random effects model using the Hartung-Knapp-Sidik-Jonkman adjustment [55] to limit the effect of studies’ diversities. According to Cohen [72], a final effect falling in the ranges of 0 to 0.2, 0.3 to 0.5, and 0.6 to 0.8 was interpreted, respectively, as small, moderate, and large. Meta-analyses were run for well-being and ill-being outcomes separately, with additional specifications of the type of outcome. In particular, we further grouped well-being and ill-being outcomes in the following dimensions: body image related, cognitive bias/flexibility, compassion, coping, mindset predisposition, mood/affect/emotions, purpose/gratitude/hope, satisfaction/quality of life, stress, and 3 funny things/3 good things. A complete list of well-being and ill-being outcomes categorized in each of the abovementioned dimensions is reported in Table 1.

The heterogeneity of the effect size was computed with the between-study variance τ^2^ and the Hartung-Knapp-Sidik-Jonkman adjustment, which allows to control for errors due to diversities in the sample sizes [73]. Heterogeneity level was interpreted as low (25%), moderate (50%), and high (75%), according to Higgins et al [74]. Potential publication biases were assessed by both funnel plots and Egger regression test for funnel plot asymmetry [75,76]. In addition, influence analyses were conducted to test if a single study accounted for a significant part of the variance in the final effect. Additional subgroup analyses were conducted to test if the effect size differed depending on the control group (waiting list vs digital control) when possible (*k*≥2 in each subgroup). To further explore the effect of age, meta-regression analyses were performed by different age categories (ie, children, adolescents, and young adults or a combination of these categories). In contrast to what we anticipated in our study protocol registered in PROSPERO, we could not run subgroup analyses to differentiate the effect of specific interventions due to the paucity of studies using the same PPIs.

**Table 1:** Well-being versus ill-being outcomes for meta-analysis.

Studies and original construct		Category	Outcome
**Krifa et al [77]**			
	Absorption	Cognitive bias/flexibility	Well-being
	Emotion regulation	Coping	Well-being
	Dedication	Mindset predisposition	Well-being
	Optimism	Mindset predisposition	Well-being
	Vigor	Mindset predisposition	Well-being
	Depression	Mood/affect/emotions	Ill-being
	Anxiety	Mood/affect/emotions	Ill-being
	Well-being	Mood/affect/emotions	Well-being
	Hope	Purpose/gratitude/hope	Well-being
	Stress	Stress	Ill-being
**Drabu et al [78]**			
	Inclination to self-injury	Cognitive bias/flexibility	Ill-being
	Pain endurance	Cognitive bias/flexibility	Well-being
	Explicit self-criticism	Cognitive bias/flexibility	Ill-being
	Implicit affect toward self	Mindset predisposition	Well-being
**Lennard et al [79]**			
	Fear of compassion from others	Cognitive bias/flexibility	Ill-being
	Fear of self-compassion	Cognitive bias/flexibility	Ill-being
	Psychological flexibility	Cognitive bias/flexibility	Well-being
	Self-compassion action	Compassion	Well-being
	Self-compassion engagement	Compassion	Well-being
	Compassion from others’ action	Compassion	Well-being
	Compassion from others’ engagement	Compassion	Well-being
	Adjustment anxiety	Mood/affect/emotions	Ill-being
	Adjustment depression	Mood/affect/emotions	Ill-being
	Breastfeeding satisfaction total	Satisfaction/quality	Well-being
	Adjustment stress	Stress	Ill-being
	Posttraumatic stress syndrome total	Stress	Ill-being
**Andersson et al [80]**			
	Self-compassion	Compassion	Well-being
	Emotion awareness/alexithymia	Mood/affect/emotions	Ill-being
	Psychological problems of clinical origin	Mood/affect/emotions	Ill-being
	Perceived stress	Stress	Ill-being
**Beshai et al [81]**			
	Self-compassion	Compassion	Well-being
	Dispositional mindfulness	Mindset predisposition	Well-being
	Nonattachment	Mindset predisposition	Well-being
	State mindfulness	Mindset predisposition	Well-being
	Anxiety	Mood/affect/emotions	Ill-being
	Depression	Mood/affect/emotions	Ill-being
	Stress	Stress	Ill-being
**Kelman et al [82]**			
	Inadequate self-compassion	Cognitive bias/flexibility	Ill-being
	Self-reassurance	Compassion	Well-being
	Affect	Mood/affect/emotions	Well-being
**Kappen et al** **[83]**			
	Partner acceptance	Mindset predisposition	Well-being
	Relationship satisfaction	Satisfaction/quality	Well-being
**Koydemir** **and** **Sun-Selışık [84]**			
	Emotional well-being	Mood/affect/emotions	Well-being
	Happiness	Mood/affect/emotions	Well-being
	Psychological quality of life	Satisfaction/quality	Well-being
	Social quality of life	Satisfaction/quality	Well-being
	Satisfaction with life	Satisfaction/quality	Well-being
**Sergeant and Mongrain [85]**			
	Maladaptive belief	Cognitive bias/flexibility	Ill-being
	Engagement	Mindset predisposition	Well-being
	Depression	Mood/affect/emotions	Ill-being
	Meaning	Purpose/gratitude/hope	Well-being
	Pleasure	Satisfaction/quality	Well-being
**Lappalainen et al [86]**			
	Psychological flexibility behavior	Cognitive bias/flexibility	Well-being
	Psychological flexibility openness	Cognitive bias/flexibility	Well-being
	Psychological flexibility value	Cognitive bias/flexibility	Well-being
	Total psychological flexibility	Cognitive bias/flexibility	Well-being
	Self-compassion	Compassion	Well-being
	Anxiety	Mood/affect/emotions	Ill-being
	Depression	Mood/affect/emotions	Ill-being
**Webb et al [87]**			
	Body image flexibility	Body image related	Well-being
	Internal body shame	Body image related	Ill-being
	External body shame	Body image related	Ill-being
	Functional body appreciation	Body image related	Well-being
	Functional body awareness	Body image related	Well-being
	Functional body satisfaction	Body image related	Well-being
	Physical activity behavior	Body image related	Well-being
	Physical activity cognitive	Body image related	Well-being
	Body appreciation	Body image related	Well-being
	Weight bias	Body image related	Ill-being
	Drive for leanness	Body image related	Ill-being
	Self-compassion	Compassion	Well-being
**Brouzos et al [88]**			
	Fear of COVID-19	Cognitive bias/flexibility	Ill-being
	Resilience	Coping	Well-being
	Empathic concern	Mindset predisposition	Well-being
	Fantasy	Mindset predisposition	Well-being
	Perspective taking	Mindset predisposition	Well-being
	Emotional loneliness	Mood/affect/emotions	Ill-being
	Positive affect	Mood/affect/emotions	Well-being
	Negative affect	Mood/affect/emotions	Ill-being
	Anxiety	Mood/affect/emotions	Ill-being
	Depression	Mood/affect/emotions	Ill-being
	Overall loneliness	Mood/affect/emotions	Ill-being
	Social loneliness	Mood/affect/emotions	Ill-being
	Personal distress	Stress	Ill-being
**Greer et al [89]**			
	Anxiety	Mood/affect/emotions	Ill-being
	Depression	Mood/affect/emotions	Ill-being
	Positive emotion	Mood/affect/emotions	Well-being
	Negative emotion	Mood/affect/emotions	Ill-being
**Tagalidou et al [90]**			
	Subjective perceived change: coping humor	Coping	Well-being
	Cheerfulness: coping humor	Coping	Well-being
	Coping humor: coping humor	Coping	Well-being
	Depression: coping humor	Coping	Ill-being
	Bad mood: coping humor	Coping	Ill-being
	Happiness: coping humor	Coping	Well-being
	Seriousness: coping humor	Coping	Ill-being
	Seriousness: 3 funny things	3 funny things/3 good things	Ill-being
	Seriousness: 3 good things	3 funny things/3 good things	Ill-being
	Coping humor: 3 funny things	3 funny things/3 good things	Well-being
	Coping humor: 3 good things	3 funny things/3 good things	Well-being
	Subjective perceived change: 3 funny things	3 funny things/3 good things	Well-being
	Subjective perceived change: 3 good things	3 funny things/3 good things	Well-being
	Cheerfulness: 3 funny things	3 funny things/3 good things	Well-being
	Cheerfulness: 3 good things	3 funny things/3 good things	Well-being
	Depression: 3 funny things	3 funny things/3 good things	Ill-being
	Depression: 3 good things	3 funny things/3 good things	Ill-being
	Bad mood: 3 funny things	3 funny things/3 good things	Ill-being
	Bad mood: 3 good things	3 funny things/3 good things	Ill-being
	Happiness: 3 funny things	3 funny things/3 good things	Well-being
	Happiness: 3 good things	3 funny things/3 good things	Well-being
**Bronk et al [91]**			
	Hope: purpose	Purpose/gratitude/hope	Well-being
	Gratitude: purpose	Purpose/gratitude/hope	Well-being
	Hope: gratitude	Purpose/gratitude/hope	Well-being
	Gratitude: gratitude	Purpose/gratitude/hope	Well-being
	Identified purpose: gratitude	Purpose/gratitude/hope	Well-being
	Identified purpose: purpose	Purpose/gratitude/hope	Well-being
	Prosocial intentions: gratitude	Purpose/gratitude/hope	Well-being
	Prosocial intentions: purpose	Purpose/gratitude/hope	Well-being
	Searching for purpose: gratitude	Purpose/gratitude/hope	Well-being
	Searching for purpose: purpose	Purpose/gratitude/hope	Well-being
**Gu et al [92]**			
	Compassion-focused theory: self-criticism	Cognitive bias/flexibility	Ill-being
	Compassion-focused theory: sensitivity to others	Cognitive bias/flexibility	Ill-being
	Compassion-focused theory: shame	Cognitive bias/flexibility	Ill-being
	Rational emotive behavior therapy: self-criticism	Cognitive bias/flexibility	Ill-being
	Rational emotive behavior therapy: sensitivity to others	Cognitive bias/flexibility	Well-being
	Rational emotive behavior therapy: shame	Cognitive bias/flexibility	Ill-being
	Rational emotive behavior therapy: tolerance of uncomfortable things	Cognitive bias/flexibility	Well-being
	Compassion-focused theory: tolerance of uncomfortable things	Cognitive bias/flexibility	Well-being
	Compassion-focused theory: compassion	Compassion	Well-being
	Compassion-focused theory: self-compassion	Compassion	Well-being
	Rational emotive behavior therapy: compassion	Compassion	Well-being
	Rational emotive behavior therapy: self-compassion	Compassion	Well-being
	Compassion-focused theory: kindness to others	Mindset predisposition	Well-being
	Compassion-focused theory: kindness to self	Mindset predisposition	Well-being
	Rational emotive behavior therapy: kindness to others	Mindset predisposition	Well-being
	Rational emotive behavior therapy: kindness to self	Mindset predisposition	Well-being
	Compassion-focused theory: anxiety	Mood/affect/emotions	Ill-being
	Compassion-focused theory: depression	Mood/affect/emotions	Ill-being
	Rational emotive behavior therapy: anxiety	Mood/affect/emotions	Ill-being
	Rational emotive behavior therapy: depression	Mood/affect/emotions	Ill-being
**Alexiou et al [93]**			
	Depersonalization	Cognitive bias/flexibility	Ill-being
	Personal accomplishment	Mindset predisposition	Well-being
	Depression	Mood/affect/emotions	Ill-being
	Anxiety	Mood/affect/emotions	Ill-being
	Emotional exhaustion	Mood/affect/emotions	Ill-being
	Positive emotions	Mood/affect/emotions	Well-being
	Negative emotions	Mood/affect/emotions	Ill-being
	Satisfaction with life	Satisfaction/quality	Well-being
	Stress	Stress	Ill-being
**Manicavasagar et al [94]**			
	Depression	Mood/affect/emotions	Ill-being
	Anxiety	Mood/affect/emotions	Ill-being
	Well-being	Mood/affect/emotions	Well-being
	Stress	Stress	Ill-being

## Results

### Overview

**Figure 1:**
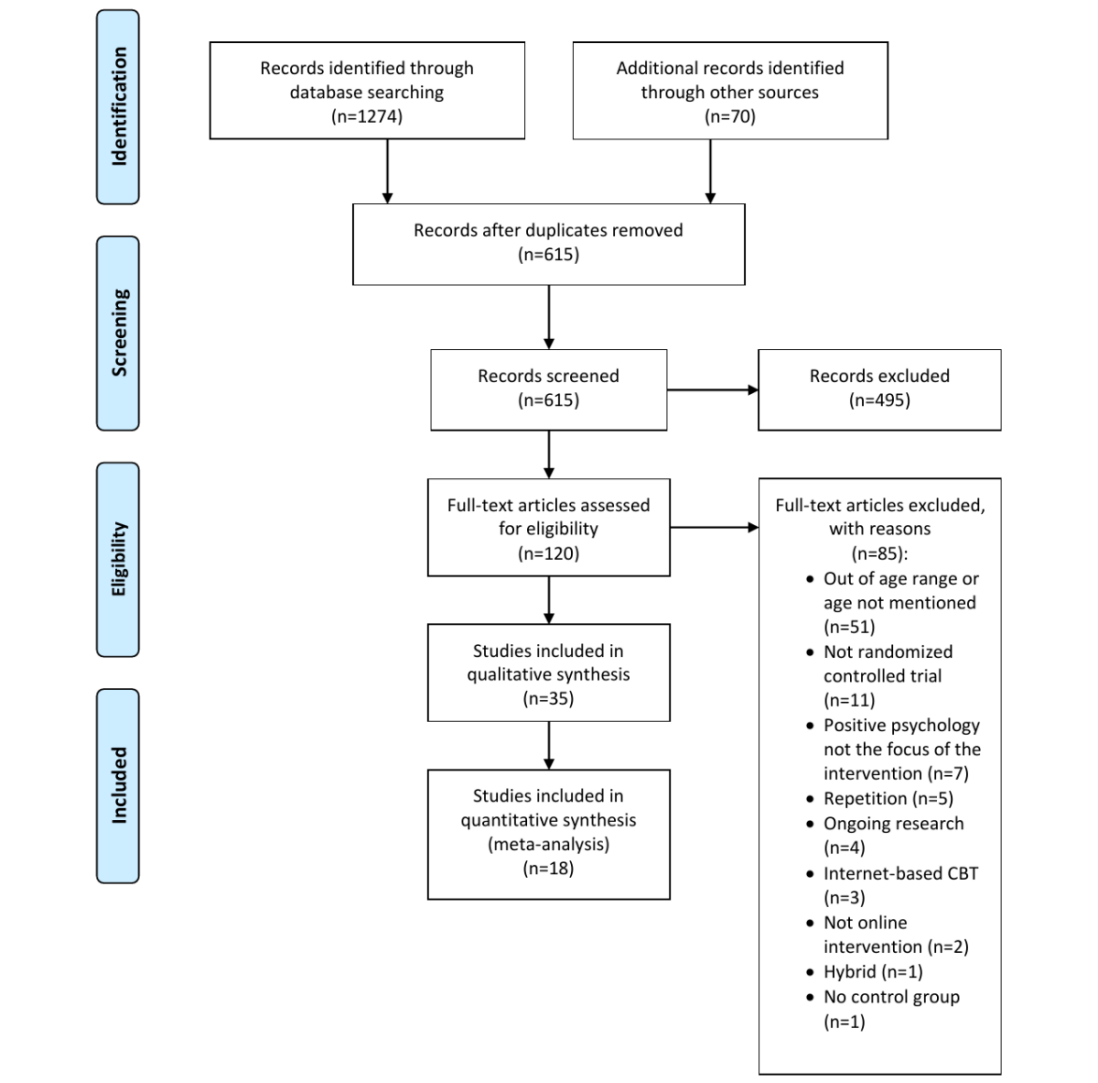
PRISMA (Preferred Reporting Items for Systematic Reviews and Meta-Analyses) flowchart of included studies in systematic review and meta-analysis [95]. CBT: cognitive behavioral therapy.

The study selection process is reported in the PRISMA flowchart (Figure 1). The initial database and hand search returned 1344 publications, of which 729 (54.24%) were duplicates, which were removed. After title and abstract screening of 615 (45.76%) records, we assessed 120 full-text articles for eligibility. We then excluded 85 (70.8%) articles, resulting in a qualitative assessment of 35 (29.2%) articles and a meta-analysis of 18 studies. The reasons for exclusion include out of age range or the age was not explicitly mentioned (n=51, 60%), positive psychology was not the main focus of the intervention (n=7, 8%), the CBT was internet based (n=3, 4%), the intervention was not web based (n=2, 2%), the trials were not RCTs (n=11, 13%) or CTs (n=1, 1%), the intervention was hybrid (n=1, 1%), repetitions (n=5, 6%), and the research was still ongoing (n=4, 5%). Cohen κ was calculated as a measure on intercoder reliability, and it was excellent (κ=0.95).

### Study Characteristics

The systematic review is based on 35 studies (Multimedia Appendix 1 [77-94,96-112]). Overall, the analytical sample amounts to 7341 participants, of which 19 studies (54%) mentioned young adults aged 20 to 35 years of age; 8 studies (23%) were focused on children, adolescents, and young adults aged up to 20 years of age; and 3 studies (9%) mentioned children, adolescents, and adults aged up to 35 years. A total of 3 studies (9%) mentioned young adults and adult participants, while 1 (3%) study mentioned children, adolescents, and adults, and 1 study (3%) mentioned all age groups, that is, children, adolescents, young adults, and adults. Gender distribution for males and females has been mentioned separately in 28 (80%) studies, while 7 (20%) studies only mentioned the percentage of female participants [79,85,87,96-99]. A total of 27 studies (77%) mentioned the ethnicity of the participants.

A total of 13 (37%) studies were conducted in Europe (Sweden, Slovakia, London, the Netherlands, Norway, Turkey, Finland, Germany, Greece, Spain, and Austria); 11 (31%) studies were conducted in North America (both United States and Canada combined); 7 (20%) in Asia (Turkey, Singapore, India, China, Japan, and South Korea); 3 (9%) in Australia, and 1 (3%) in Africa (Tunisia). The duration of the interventions varied from 1 week to a maximum of 12 weeks. Of all the studies, 23 (66%) studies had follow-up assessments. In particular, 19 (54.3%) studies had 1 follow-up, 2 (6%) studies had 2 follow-ups, and 2 (6%) had 3 follow-ups (Table 2). The duration of the follow-ups ranged from 2 to 12 weeks. The range of the interventions was 11 weeks (from 1 to 12 weeks), while the median duration was 12 weeks.

Data from the participants were collected through web-based surveys and questionnaires as well as in person. A total of 26 (74%) studies used convenience sampling techniques, and 7 (20%) studies used purposive sampling procedures. In 1 (3%) study, experience sampling was used, while in 1 (3%) study, the details of sampling were not clearly mentioned; 29 (83%) studies conducted the trial on the general population (nonclinical), while 6 (17%) studies conducted the trial on clinical population (ie, individuals who engaged in nonsuicidal self-injury, scored high on scales of depression, had anxiety and stress, had mental disorder or psychological stress, had autism spectrum disorder, were undergoing active cancer treatment). A total of 28 (80%) studies conducted the intervention in a web-based setting. In addition, 4 (11%) studies conducted the intervention through smartphone-based applications or SMS text messaging; 1 (3%) study used a hybrid setting, and 1 (3%) conducted the intervention in a telehealth setting [100,101]. Interestingly, 1 (3%) study conducted the intervention through Instagram, and 1 (3%) study used the Vivibot chatbot [89,102].

**Table 2:** Study characteristics.

Study	Study design	Duration of intervention	Follow-up and timing of follow-up	Experimental group, n	Control group, n	Characteristics of control group	Sampling
Mahalik et al [112]	RCT^a^	—^b^	—	Psychoeducation=61, psychoeducation and purpose reflection=70	52	Waitlist control	—
Krifa et al [77]	2-armed RCT, pretest and posttest	8 weeks	1 (12 weeks)	183	183	Waitlist control	Convenience sampling
Drabu et al [78]	RCT, pretest and posttest	T1-postsession one; post one- week training-T2	1 (2 weeks after completion of second session)	30	33	Digital control	Convenience and purposive sampling
Lennard et al [79]	RCT	—	1 (8 weeks)	231	239	Waitlist control	Convenience sampling
Andersson et al [80]	RCT, pretest and posttest	6 weeks	—	Compassion and mindfulness group=25 each	15	Waitlist control	Convenience sampling
Beshai et al [81]	RCT, pretest and posttest	4 weeks	—	227	229	Digital control	Convenience sampling
Chilver and Gatt [96]	RCT, pretest and posttest	6 weeks	1 (7 weeks)	205	204	Digital control	Convenience sampling
Hussong et al [104]	RCT, pretest and posttest	1 week (parents asked to complete the program within the week)	1 (4 weeks)	53	51	Waitlist control	Convenience and purposive sampling
Halamová et al [105]	RCT, pretest and posttest	2 weeks (14 days)	1 (8 weeks)	70	53	Waitlist control	Convenience sampling through snowballing technique
Kelman et al [82]	RCT, pretest and posttest	2 weeks	1 (2 weeks)	69	68	Digital control	Convenience sampling
Hamm et al [97]	RCT, pretest and posttest	4 weeks (1 month)	3 (12 weeks)	628	628	Waitlist control	Convenience sampling
Daugherty et al [98]	Quasi-experimental, RCT, pretest and posttest	1 month (28 days)	—	66	46	Digital control	Convenience sampling
Halamová et al [108]	RCT, pretest and posttest	2 weeks (14 days)	1 (8 weeks)	69	53	Waitlist control	Convenience sampling
Kappen et al [83]	RCT, pretest and posttest	2 weeks (12 days)	—	56	57	Digital control	Convenience sampling
Galante et al [99]	RCT, pretest and posttest	4 weeks	—	409	400	Digital control	Convenience sampling
Halamová et al [109]	RCT, pretest and posttest	2 weeks (15 days)	1 (8 weeks)	93	53	Waitlist control	Convenience sampling
Drozd et al [110]	RCT	4 weeks	3 (4 weeks, 8 weeks, and 24 weeks)	112	94	Waitlist control	Convenience sampling
Koydemir and Sun-Selışık [84]	RCT	8 weeks	—	48	44	Waitlist control	Convenience sampling
Sergeant and Mongrain [85]	RCT	3 weeks	2 (4 weeks and 8 weeks)	253	213	Digital control	Convenience sampling
Lappalainen et al [86]	RCT, pretest and posttest	5 weeks	—	iACT^c^ student coach+virtual coach group =116 and iACT virtual coach group=116	116	Waitlist control	Convenience sampling
Tay [106]	RCT, pretest and posttest	2 weeks	1 (8 weeks)	97	78	Digital control	Convenience sampling and purposive sampling
Paetzold et al [100]	RCT, pretest and posttest	6 weeks	1 (4 weeks)	46	46	Waitlist control	Experience sampling
Qu et al [101]	RCT	12 weeks	—	Program evaluation=56.25%; focus group interview=70.8%	Program evaluation=43.75%; focus group intervein=29.2%	Digital control	Convenience sampling and snowball sampling
Webb et al [87]	RCT, pretest and posttest	4 weeks	1	159	129	Waitlist control	Purposive sampling
Nawa and Yamagishi [103]	RCT, pretest and posttest	2 weeks	2 (4 weeks and 12 weeks)	42	42	Digital control	Convenience sampling
Brouzos et al [88]	Quasi-experimental, pretest and posttest	2 weeks	1 (2 weeks)	44	38	Not explicit	Convenience sampling
Pizarro-Ruiz et al [107]	RCT, pretest and posttest	2 weeks=14 days	—	89	75	Digital control	Convenience sampling
Halamová et al [111]	RCT, pretest and posttest	13 days=2 weeks	1 (8 weeks)	91	53	Waitlist control	Convenience sampling
Sampson et al [102]	RCT, pretest and posttest	Recruitment: 5 months=20 weeks	—	71	61	Digital control	Convenience sampling
Greer et al [89]	RCT	4 weeks=28 days	1 (8 weeks)	25	20	Waitlist control	Convenience and snowball sampling
Tagalidou et al [90]	RCT, pretest and posttest	1 week	1 (4 weeks)	Coping humor=35, three funny things=46, three good things=52	Early memories=49	Waitlist control	Convenience sampling
Bronk et al [91]	RCT, pretest and posttest, and lagged posttest	3 days	1 (1 week)	Gratitude condition=74; purpose condition=79	71 in the control condition	Waitlist control	Convenience sampling
Gu et al [92]	RCT, pretest and posttest	4 weeks	1 (2 weeks)	CFI^d^=10 and REBT^e^=10	12	Waitlist control	Convenience sampling
Alexiou et al [93]	RCT, pretest and posttest	3 weeks	1 (4 weeks)	19	19	Digital control	Purposive and convenience sampling
Manicavasagar et al [94]	RCT, pretest and posttest	6 weeks	—	120	115	Digital control	Convenience sampling

^a^RCT: randomized controlled trial.

^b^Not applicable.

^c^iACT: internet-based acceptance and commitment therapy.

^d^CFI: compassion-focused therapy-based intervention.

^e^REBT: rational emotive behavior therapy.

### Theoretical Background

The broaden-and-build theory was the widely used theory in 5 (14%) studies [84,89,91,99,103]. The broaden-and-build theory of positive emotion states that certain discrete positive emotions, for example, joy, contentment, pride, and love, share the ability to broaden momentary thought-action repertoires of people and build their enduring personal resources, ranging from physical and intellectual resources to social and psychological resources [113]. Other studies frequently mentioned in the articles included acceptance and commitment theory [79] (oriented toward the development of psychological flexibility), affect theory [80] (emotions generating weak or strong ties to relations), attachment theory [80,92,100] (individuals born with innate behaviors function to attract proximity to attachment figures), Eisenberg’s theory of parent emotion socialization [104] (parents’ emotion socialization behaviors driving children’s emotion socialization), emotion-focused therapy theory [105] (integrating person-focused care with modern emotion theory), motivational theory of life span development (Heckausen’s theory) [97] (role of individual in lifespan development), Bandura’s self-efficacy theory [106] (belief that one can execute needed steps to achieve a goal), social mentality theory [92,100] (both care-seeking and caregiving mentalities are activated when one is being self-compassionate and reassuring), embodiment theory [87] (psychological processes influenced by body), self-determination theory [103] (internalizing regulation and self-regulation), theory of mindfulness [107] (being actively engaged is beneficial for a rigid and judgmental mindset), Festinger’s social comparison theory [102] (based on social comparison), stress and coping theory [89] (coping with stressful situations), and humor theory [90] (cognitive view of humor).

### Quality Assessment

Among all 35 articles, the summary score of the quality assessment ranged from 12 [78] to 29 [77,102], with a median of 22.5 points. Among all criteria, most of the papers (31/35, 89%) did not report all important harms or unintended effects in each group and protocol of the full trial. Blinding was either not performed or not explicitly reported by 86% (n=30) of the studies. Also, most papers (26/35, 74%) lacked more detailed information about any changes to trial outcomes after the trial started with reasons, and 24 (69%) studies lacked information regarding the mechanism used to implement the random allocation sequence (such as sequentially numbered containers) and the description of any steps taken to conceal the sequence until interventions were assigned; 23 (66%) studies did not report explanation of any interim analyses and stopping guidelines, and 22 (63%) studies did not mention essential changes to methods after trial commencement (such as eligibility criteria) with reasons. Finally, 21 (60%) studies did not calculate both absolute and relative effect sizes for binary outcomes. A detailed description of each study evaluation is reported in Multimedia Appendix 2 [77-94,96-112].

### PPIs Used in the Studies

A diverse range of PPIs were conducted among the study participants and is reported in detail in Multimedia Appendix 3 [77-94,96-112].

#### Web-Based Meditation and Mindfulness

Drabu et al [78] used web-based, self-compassion–based, guided meditation for nonsuicidal self-injury. Krifa et al [77] experimented with a web-based multicomponent intervention that included lectures, expert videos, psychoeducation, and positive psychology practices to assist Tunisian students with mental health. Kelman et al [82] compared web-based compassion mind training and CBT for perinatal and pregnant women. Beshai et al [81] used web-based psychoeducational videos, guided meditations, and exercises to reduce anxiety and depression. Halamová et al [105,108,109] studied self-compassion and self-criticism through various web-based and smartphone-assisted exercises. Pizarro-Ruiz et al [107] conducted guided mindfulness sessions via a smartphone app (Aire Fresco). Gu et al [92] experimented on Chinese students to mitigate depression and anxiety through web-based individual counseling sessions regarding mindfulness meditation (MP3 files).

#### Positive Psychology and Self-Compassion

Lennard et al [79] provided web-based self-compassion training for mothers. Drozd et al [110] experimented with an internet-based program, “Better Days,” which included psychoeducational exercises to increase happiness. Halamová et al [111] explored emotion-focused training and loving-kindness meditation. Galante et al [99] practiced loving-kindness meditation through web-based videos. Webb et al [87] used a web-based yoga program to improve body image satisfaction and self-compassion. Nawa and Yamagishi [103] assessed academic motivation using journal writing and web-based self-assessments, including gratitude and other daily life aspects. Brouzos et al [88] tested the “Staying Home—Feeling Positive” web-based PPI during the COVID-19 pandemic. Andersson et al [80] provided compassion mindset intervention training via smartphone app among university students. Alexiou et al [93] assessed burnout and depression among Greek health care professionals by conducting a PPI. Manicavasagar et al [94] used “Bite Back,” a multicomponent web-based positive psychology to increase well-being among young adults.

#### Gratitude and Acts of Kindness

Chilver and Gatt [96] explored self-compassion and acts of kindness through web-based modules. Hussong et al [104] examined parent-child gratitude conversations using web-based tools. Tagalidou et al [90] used web-based humorous diary writing techniques to address happiness and depression.

#### Optimism and Positive Emotion

Sergeant and Mongrain [85] analyzed optimism among participants through web-based diary writing exercises. Tay [106] assessed a web-based Hope, Optimism, and Positive Emotion intervention. Sampson et al [102] used Instagram images to assess body, facial, and smile dissatisfaction. Koydemir and Sun-Selışık [84] analyzed optimism among participants by using alternating web-based diary writing exercises. Hamm et al [97] focused on improving goal engagement and optimism among university students.

#### Relationship Satisfaction and Acceptance

Kappen et al [83] assessed relationship satisfaction and partner acceptance through web-based psychoeducation. Qu et al [101] analyzed sensory social routines, attention, dyadic engagement, and nonverbal communication in children with autism using synchronous group-based parent coaching sessions via telehealth.

#### Purpose and Well-Being

Mahalik et al [112] assessed the Father Project webpage’s intervention for fathers’ sense of purpose. Paetzold et al [100] aimed to enhance resilience through web-based ecologic momentary interventions and face-to-face sessions. Lappalainen et al [86] used ACT intervention to increase self-compassion skills and psychological flexibility during the COVID-19pandemic. Bronk et al [91] conducted the Purpose Toolkit and Gratitude Toolkit to increase a sense of purpose among participants. Greer et al [89] studied psychological well-being among patients with cancer using Vivibot, a chatbot designed to deliver positive psychological skills. Daugherty et al [98] used a smartphone app for hope and well-being.

### Digital Control Versus Waitlist Control

The control groups were categorized into 2 groups: digital controls (15/35, 43%) and waitlist controls (19/35, 54%; Table 2). Digital controls involved some form of digital or web-based interaction but did not include the full intervention content. They had access to certain activities or features but did not receive the complete intervention that the experimental group received. For example, digital control groups included audio recording; video watching; internet-based communication [78,81,82]; psychoeducation; web-based daily registry of relationship experiences; web-based diary writing activities without positive psychology components; and digital placebo activities (writing daily events, early memories, and life events) [83,85,93,94]. Other control measures included elements of positive psychology that differed from the focus of the intervention (the digital control group had identical initial app assessment as the intervention group but did not receive the complete treatment or they had access to a website featuring inspirational phrases) [96,98,99,106]. In other cases, the control group even performed daily self-evaluations without an equivalent active task, downloaded the Lumosity smartphone app, or used neutral Instagram images of nature [101-103,107].

The waitlist controls referred to the control groups in which participants did not receive the active intervention during the initial phase of the study but were promised or scheduled to receive it at a later time. The waitlist control group participants do not receive the full intervention immediately and instead are placed on “waitlist” to receive the intervention after a specified waiting period. RCT control groups either received no treatment or were given access to full digital intervention content after the trial. Among the 35 studies, 1 (3%) study did not explicitly mention the category of the control group [88].

### Outcomes of PPIs

Outcomes were related to both ill-being and well-being components (Table 1). In particular, 27% (15/18) of studies focused on ill-being, among others prominently including depression (11/15, 73%), anxiety (9/15, 60%), stress (8/15, 53%), and loneliness (3/15, 20%) using measures such as the Depression, Anxiety, and Stress Scales, the Generalized Anxiety Disorder scale, the short and long forms of the Spielberger State-Trait Anxiety Inventory, and De Jong Gierveld Loneliness Scale.

Well-being outcomes included compassion (6/18, 33%), satisfaction (7/18, 39%), optimism (1/18, 6%), happiness (3/18, 17%), resilience (1/18, 6%), emotion regulation and emotion awareness (6/18, 33%), hope (3/18, 17%), mindfulness (2/18, 11%), purpose (1/18, 6%), quality of life (1/18, 6%), gratitude (1/18, 6%), empathy (1/18, 6%), forgiveness (1/18, 6%), motivation (1/18, 6%), and kindness (1/18, 6%) using the Self‐Compassion Scale (Self‐Compassion Scale‐Short Form), Satisfaction with Life Scale, Life Orientation Test-Revised, Authentic Happiness Inventory, Connor-Davidson Resilience Scale, Profile of Emotional Competence, Snyder Hope scale, The Five Facet Mindfulness Questionnaire-15, Claremont Purpose Scale, “Psychological Health” and “Social Relationships” subscales of WHO Quality of Life-Brief Version, Gratitude Questionnaire, Interpersonal Reactivity Index, Heartland Forgiveness Scale, and Chinese Compassion Scale.

Both ill-being and well-being included components of self-criticism and self-reassurance (6/18, 33%), well-being (both positive and negative; 5/18, 28%), positive and negative effect (4/18, 22%) using Forms of Self-Criticism and Reassuring Scale, Warwick-Edinburgh Mental Well-being Scale and Positive and Negative Affect Scale.

### Meta-Analytic Results

**Figure 2:**
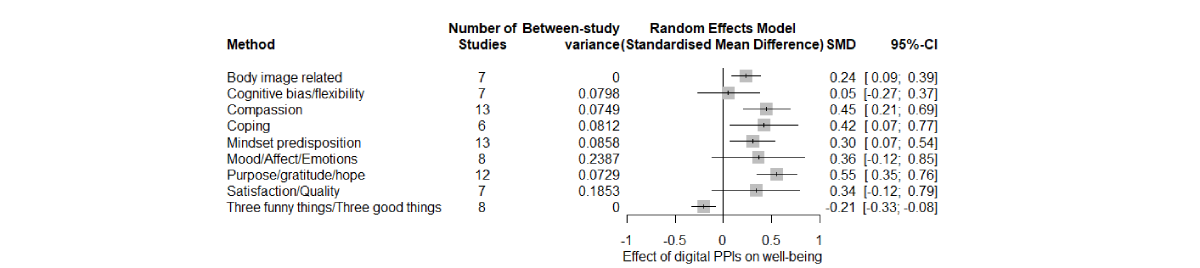
Meta-analyses on well-being outcomes for positive psychology interventions (PPIs).

Meta-analyses on well-being outcomes showed that PPIs improved purpose, gratitude, and hope with a medium-to-large effect size (*k*=12; Hedges *g*=0.555, 95% CI 0.348-0.761; *P*<.001; *I*^2^=70%). Only 1 (%) study involved a digital control group, for which the reported effect was significantly smaller (Hedges *g*=0.09). In addition, PPIs augmented the levels of compassion (*k*=13; Hedges *g*=0.447, 95% CI 0.210-0.684; *P*=.001; *I*^2^=62%), with no significant differences (*P*=.34) between the waiting list (*k*=11; Hedges *g*=0.356) and the digital control group (*k*=2; *g*=0.670). In addition, PPIs augmented the positive coping behaviors (*k*=6; Hedges *g*=0.421; 95% CI 0.072-0.770; *P*=.003; *I*^2^=72%) with a medium effect size. PPI interventions also improved body image–related outcomes with a medium effect (*k*=7; Hedges *g*=0.238, 95% CI 0.090-0.388; *P*=.007; *I*^2^=0%). A small-to-medium effect was found for mindset predisposition (*k*=13; Hedges *g*=0.304, 95% CI 0.072-0.537; *P*=.02; *I*^2^=74%), with a significant difference between the control groups (*P*=.01). In particular, effect size was larger and significant when a waiting list (*k*=6; Hedges *g*=0.534) was included as the control group when compared with the digital controls (*k*=7; Hedges *g*=0.092). Also, a small-to-medium effect was also found for the variable 3 funny things/3 good things (*k*=8; Hedges *g*=–0.206, 95% CI –0.328 to –0.083; *P*=.005; *I*^2^=0%) with all studies including the waiting list control groups. A nonsignificant effect was found for cognitive flexibility (*k*=7; Hedges *g*=0.054, 95% CI –0.265 to 0.372; *P*=.69; *I*^2^=75%) and mood/affect/emotions (*k*=8; Hedges *g*=0.364, 95% CI –0.120 to 0.849; *P*=.12; *I*^2^=81%), the latter with no difference in the control groups (*P*=.40) although participants in the waiting list showed a larger effect size (*k*=5; Hedges *g*=0.570) when compared with the digital control (*k*=3; Hedges *g*=0.088). Also, satisfaction and quality of life (*k*=7; Hedges *g*=0.338, 95% CI –0.119 to 0.793; *P*=.12; *I^2^*=81%) showed a nonsignificant effect, with no differences between subgroups (*P*=.65; Figure 2).

**Figure 3:**
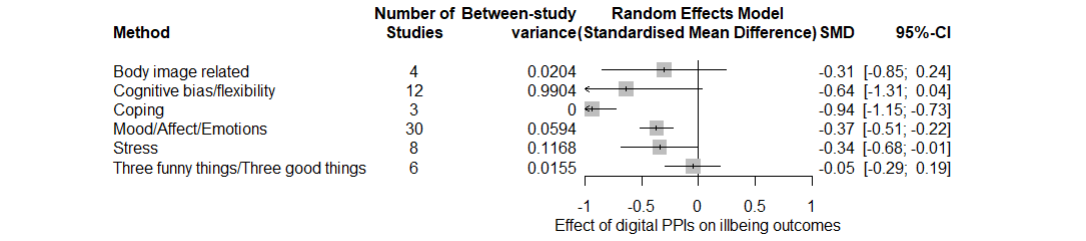
Meta-analyses on ill-being outcomes for positive psychology interventions (PPIs).

Ill-being outcomes were less represented in the included studies (Figure 3). Meta-analyses showed a large negative effect for the reduction of cognitive biases (*k*=14; Hedges *g*=–0.637, 95% CI 1.309 to –0.036; *P*=.05; *I*^2^=94%), with no group differences between the waiting list and the digital control group (*P*=.54), although once again effect sizes tended to be larger when a waiting list (*k*=7; Hedges *g*=–0.799) was considered with respect to digital control groups (*k*=5; Hedges *g*=–0.405). PPIs showed a medium-to-large effect on the reduction of negative emotions and mood problems (*k*=30; Hedges *g*=–0.369, 95% CI –0.513 to –0.225; *P*<.001; *I*^2^=60%). Interestingly, subgroup differences showed that the effect size was significantly (*P*=.03) larger for studies including a waiting list (*k*=21; Hedges *g*=–0.456) when compared with studies including digital control groups (*k*=9; Hedges *g*=–0.200). PPIs also diminished stress levels (*k*=8; Hedges *g*=–0.342, 95% CI –0.677 to –0.007; *P*=.045; *I*^2^=81%), with no significant differences in the effect size (*P*=.35) between studies including a waiting list (*k*=5; Hedges *g*=–0.441) versus digital control groups (*k*=3; Hedges *g*=–0.157). A very large effect was found for coping (*k*=3; Hedges *g*=–0.939,95% CI –1.151 to –0.728; *P*=.003; *I*^2^=0%); however, interpretation of this result would be limited due to the low number of studies. While the effect sizes were not significant for body image–related outcomes (*k*=4; Hedges *g*=–0.305, 95% CI –0.851 to –0.240; *P*=.17; I^2^=17%) and 3 funny things/3 good things (*k*=6; Hedges *g*=–0.048, 95% CI –0.289 to –0.192; *P*=.63; *I*^2^=28.5%).

**Figure 4:**
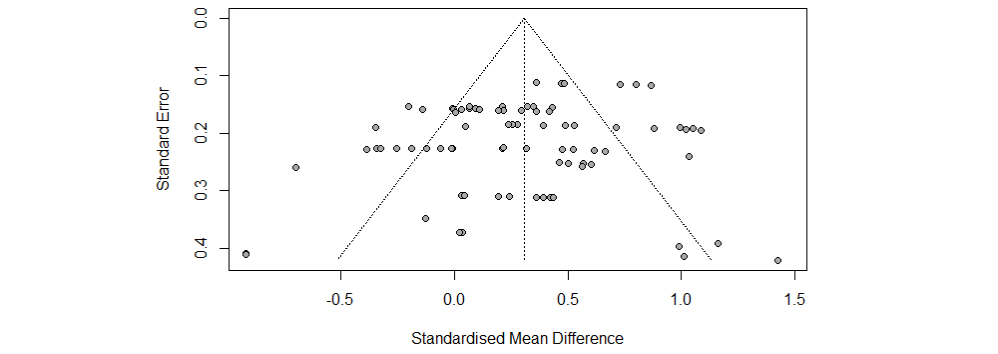
Funnel plot of well-being outcomes for positive psychology interventions.

**Figure 5:**
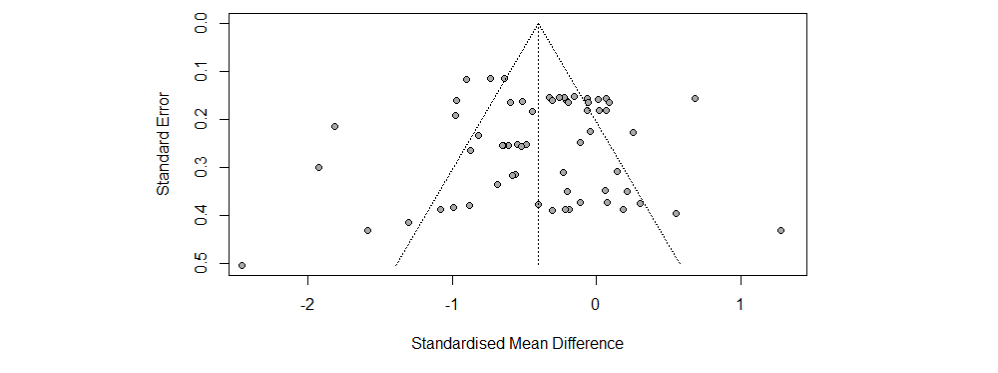
Funnel plot of ill-being outcomes for positive psychology interventions.

Funnel plots were symmetrical for the overall meta-analysis of both the well-being and ill-being outcomes (Figures 4 and 5), and the regression test for funnel plot asymmetry was not significant in both cases as well, thus confirming the absence of publication biases. Influence analyses revealed that that a single study did not account for a significant part of the variance in the final effect.

Finally, meta-regression analyses showed that PPIs tended to show a larger effect size on well-being outcomes in studies including young adults (β=.322; *P*=.008), while no specific effect was found for ill-being outcomes. Figures S1 and S2 in Multimedia Appendices 4 and 5 report additional detailed information of each meta-analysis.

## Discussion

### Principal Findings

In our systematic review and meta-analysis of 35 studies and 18 studies, respectively, we examined the impact of digital interventions grounded in positive psychology on the well-being and ill-being of children, adolescents, and young adults. Our results showed 4 main findings. First, when it comes to well-being outcomes, PPIs enhanced various facets of well-being, notably purpose, gratitude, and hope, with a medium-to-large effect, as well as compassion, positive coping (eg, coping with humor), and body image–related concerns with a medium effect. Smaller effects were found for mindset predisposition and 3 funny things/3 good things, while PPIs did not seem to improve mood and positive emotions, satisfaction and quality of life, and cognitive flexibility. This aligns with prior investigations of PPIs in traditional settings [17]. These interventions seem to provide robust support in enhancing aspects of well-being that involve an individual’s outlook on life and their ability to foster a sense of personal achievement and satisfaction.

Second, when we looked at ill-being outcomes, the picture was different. In particular, the larger effect was found for diminishing cognitive biases, including self-criticism and fears, followed by a decrement in negative emotions and mood problems, especially when participants of the experimental group were compared with the waiting list. Hence, PPIs can be a useful tool in reducing cognitive biases typical of, for example, mood problems, and stress levels [83]. To note, it is crucial to differentiate the control groups in the analyses. Indeed, although we could not make subgroup comparisons for all the outcomes due to the paucity of studies in each group, we showed that effects sizes tended to be consistently larger in studies including a waiting list rather than a digital control group (eg, including some sort of web-based interactions). Digital control groups, such as those engaging in nonspecific digital activities or using general health apps, could serve as valuable benchmarks. This would allow us to distinguish the specific contributions of PPIs from broader digital engagement effects. Such comparisons would shed light on the specific psychological mechanisms activated by PPIs compared with general digital exposure, helping to isolate the unique elements of PPIs that contribute to improved well-being outcomes.

Third, several studies within our review highlighted the efficacy of interventions tailored to specific settings and contexts. For example, digital interventions aimed at fostering hope and optimism were found to be particularly beneficial for college students prone to failure and those with low optimism levels [63]. Also, interventions focusing on self-compassion were found to be especially beneficial for mothers of infants, offering them a respite from the unique challenges of early parenthood [61]. By contrast, interventions that used smartphone delivery, such as the hope intervention, showcased the adaptability and accessibility of digital platforms, making well-being practices more integrated into daily routines [64]. Another noteworthy finding was the positive impact of multicomponent PPIs delivered web-based for subjective well-being of young adults [62].

Fourth, when age was considered as a moderator, studies with young adult participants showed larger effect sizes in the meta-analysis with well-being outcomes, but no differences emerged with respect to ill-being indicators. This is an important consideration since young adults might be more inclined to understand the importance of promoting well-being and thus more willing to take part in interventions and experiencing the positive effects. However, while some demographic groups appeared to benefit more from certain types of interventions, the overall evidence was not strong enough to conclusively determine that these effects were consistently replicated across different age groups, such as children, adolescents, or young adults [17,31].

Finally, the variability in intervention efficacy highlights the critical role of intervention design and implementation in achieving desired outcomes. This variation underscores the need for carefully tailored interventions that consider the unique needs and circumstances of the target demographic to optimize efficacy. Therefore, while PPIs hold promise, the evidence suggests that a nuanced approach to their application is necessary, where factors such as intervention type, target population, and desired well-being outcome are all carefully considered to maximize benefits [17,31].

When compared with the findings of existing literature, our findings provide a nuanced view that aligns with some previous studies but also highlights the complexity of applying PPIs across diverse populations and settings [6,114,115]. Unlike some optimistic narratives, our results suggest that while PPIs can be beneficial, their efficacy is not universal and depends on specific intervention types and target populations. Furthermore, our findings diverge from studies such as the MYRIAD trial [116], underscoring the need for cautious interpretation of PPI efficacy and the potential for adverse effects.

### Future Directions

The findings from this systematic review and meta-analysis provide a solid foundation for understanding the effectiveness of PPIs in young populations. However, as with all research, there are avenues that remain unexplored and warrant further investigation. One primary recommendation is to conduct more rigorous RCTs with larger and more diverse samples. This would not only enhance the generalizability of the findings but also allow for a more in-depth exploration of the nuances and specific components of the interventions that are most effective. Also, we suggest that PPIs should be integrated in interventions that also collect biological information to further assess their efficacy.

Another crucial area for future research is the examination of the long-term effects of these digital interventions. While our review captured the immediate and short-term benefits, understanding the sustainability of these positive outcomes over extended periods is essential. This would provide insights into whether these interventions lead to lasting changes in well-being and mental health or whether periodic “booster” sessions are required to maintain the benefits. In addition, given the rapid advancements in technology, exploring the integration of emerging technologies, such as virtual reality or augmented reality, into PPIs could offer innovative ways to engage and support adolescents.

From a practical implementation perspective, stakeholders in the field of youth mental health should consider incorporating evidence-based digital interventions into broader mental health programs and curricula. Schools, community centers, and mental health organizations can benefit from these scalable and accessible tools, especially in regions where traditional face-to-face interventions might be limited. Collaborations between researchers, technologists, and educators can further refine and optimize these interventions, ensuring that they remain relevant and effective in the ever-evolving digital landscape.

### Limitations

Our study, while extensive, exhibits limitations that warrant attention for a comprehensive understanding of the scope and applicability of our findings. One major limitation is the heterogeneity in study settings and targeted age groups, which ranged from school environments to clinical settings and included diverse demographic categories from children to young adults [30]. This wide variability complicates the task of uniformly generalizing the results across different settings and age demographics. In addition, the medium-to-low quality of some included studies potentially undermines the reliability and robustness of our findings. The varying methodological rigor and potential biases in the study design across the analyzed studies necessitate a cautious interpretation of the effectiveness and applicability of PPIs based on this evidence base. We did not divide the results or their interpretation between interventions aiming at preventing versus treating mental health problems since our aim was to explore the literature and treatment effects of PPIs in general; however, we do acknowledge that the absence of improvement in a prevention intervention may not be the evidence that an intervention is ineffective; hence we should consider this interpretation to avoid biasing the findings of the meta-analysis. Hence, we suggest that future studies should look more carefully at this differentiation.

In addition, an important aspect that was not covered in our review is the assessment of the safety and potential adverse effects of PPIs. Not including an evaluation of harms, as highlighted by the findings from larger trials such as the MYRIAD trial, which documented no significant effects and even potential harm in certain subgroups, poses a noteworthy gap in our analysis [117]. This aspect highlights an area for further investigation, particularly considering the intricate nature of mental health interventions and their varied effects across diverse individuals. In addition, the absence of data from lower-middle-income countries and the lack of studies not published in languages other than English limit the generalizability of our conclusions globally, raising concerns about the effectiveness and safety of PPIs in these regions where cultural, economic, and health care contexts may differ significantly from those in high-income countries [30,31]. Finally, although we calculated the intercoder reliability for the screening process, we were not able to provide a measure of reliability for the quality assessment of the studies; hence, we encourage future studies to consider conducting the assessment blind and calculate a measure of agreement.

### Conclusions

In conclusion, our systematic review suggests that while PPIs can enhance certain aspects of well-being among children, adolescents, and young adults, the effects are not consistent across all domains or demographic groups. The evidence supports the effectiveness of specific types of PPIs, particularly those that enhance gratitude, purpose, and hope. However, these benefits are not uniform, and the impact varies by the type of well-being outcome and the population segment. Moreover, given the significant variability in the intervention settings, the diversity of outcomes, and the medium-to-low quality of the studies reviewed, any conclusions about the efficacy of PPIs should be viewed as tentative. The findings underscore the necessity for further rigorous research to better understand the mechanisms and effectiveness of PPIs, assess their safety, and evaluate their applicability in different geographical and clinical contexts. Future studies should also explore how digital platforms might uniquely influence the success of these interventions and consider the theoretical underpinnings of PPIs in more depth to enhance their practical and academic contributions.

## Appendix

Multimedia Appendix 1 Basic characteristics.

Multimedia Appendix 2 Quality assessment.

Multimedia Appendix 3 Characteristics of the positive psychology intervention.

Multimedia Appendix 4 Additional detailed information of each meta-analysis (Figure S1).

Multimedia Appendix 5 Additional detailed information of each meta-analysis (Figure S2).

## Abbreviations

CBT: cognitive behavioral therapy
CONSORT: Consolidated Standards of Reporting Trials
CT: controlled trial
PPI: positive psychology intervention
PRISMA: Preferred Reporting Items for Systematic Reviews and Meta-Analyses
RCT: randomized controlled trial
