# Supplementary material for: Application of Positive Psychology in Digital Interventions for Children, Adolescents, and Young Adults: Systematic Review and Meta-Analysis of Controlled Trials
Source: JMIR Ment Health. 2024 Aug 14;11:e56045. doi: 10.2196/56045 (PMC11358669; doi:10.2196/56045)
Supplement: Multimedia Appendix 1 [file mental_v11i1e56045_app1.docx]

| **Study** | **Country** | **Population** | **Ethnicity/** | **Gender** | **Age (Mean (SD), Min, Max)** |
| --- | --- | --- | --- | --- | --- |
| Mahalik et al (2022) | USA | General (Fathers) | White (77.60%), Asian or Asian American (8.74%), Black or African American (8.20%), Other race (3.28%), American Indian or Alaska Native or Native Hawaiian (1.09%) and Biracial (1.09%). | Men straight (96.72%; 1.64% gay; .55% other; and 1.09% did not report) | 33.83 (6.66), Min = 20, Max = 49 |
| Krifa et al (2022) | Tunisia | General (Healthcare Students) | 100% Tunisian | Female=94% and Male=6% | 20.74 (1.64), Min = 18, Max = 30 |
| Drabu et al (2022) | Singapore | Clinical (Engaged in non-suicidal injury) | Chinese (85.7%), Indian (3.2%), Caucasian (4.8%), Other (6.3%) | Female (71.4%) Male (25.4%) Other—undisclosed (3.2%) | 23.24 (4.65) |
| Lennard et al (2021) | Australia/New Zealand | General (Mothers) | Australian (85.0%), Asian (3.6%), European (2.8%), or New Zealander (3.2%) (other: 5.4%). | 100% Females | 32.56 (3.96) |
| Andersson (2021) | Sweden | General (University Students) | 100 % Swedish | Female: 68.4%, Male: 29.8%,  Undisclosed: 1.75% | Compassion= 23.7 (3.0); Mindfulness = 25.9 (6.0); Control= 26.6 (6.0) |
| Beshai (2020) | USA and other | Clinical (Measuring high on anxiety, depression, and stress) | Western European: (42.1%), Eastern European: (22.8%); Chinese: (3.1%); Latin American (9%); Other: (22.6%) | Female (43.9%) Non-binary (0.4%); | 35.13 (10.57) |
| Chilver and Gatt (2022) | Australia | General (Psychology students) | N/A | Females: 70% | 19.7 (3.2) |
| Hussong et al (2020) | USA | General (Families) | 80% White, 5% Latinx, 5% African American, 9% Asian, 1% American Indian or Alaska Native, and 5% Middle Eastern. | Children: 50% Female. Parents: Female (86%, Mothers and 14% Fathers) | Children 10.8 (1.17), Min = 9, Max = 13. Parents 40.8 (5.18) |
| Halamova et al (2018) | London/Slovakia | General (Community) | 100% Caucasian and Slovaks | Female: 82.7%, Male: 15.6% | Control Group = 25.35 (6.32). Intervention Group = 33.73 (16) |
| Kelman et al (2018) | USA and India | General (Pregnant or postpartum) | African American: (8.3%). Asian: (28.6%). Southeast Asian: (1.2%). European American/White: (53.6%). Latino/a: (6%). Others: (2.4%). USA: (78.6%). India: (21.4%) | Heterosexual: (88.1%). LGBTQ: (7.1%). Neither describes me: (4.8%) | 18-25 years: 19 (22.6); 26-34 years: 52 (61.9); 35-54 years: 13 (15.5)  Min= 18 Max = 54 |
| Hamm et al (2019) | Canada | General (University students) | N/A | Females: 63% | 80% of students were Min= 17 Max= 20 |
| Daugherty et al (2018) | USA | General (Undergraduate Student Volunteers) | (88.4%) identified as White Non-Hispanic. | Females: 70.5% | Min= 18 Max= 25 |
| Halamova et al (2020) | Slovakia | General (Community) | N/A | Intervention Group:  Females: 79.4%  Males: 20.5%  Control Group:  Females: 85% and Males: 15% | Intervention Group: 32.24 (10.44)  Control Group: 25.35 (6.32) |
| Kappen et al (2019) | Netherlands | General participants | N/A | Female:  Intervention Group: 86%  Control Group: 93%  Male:  Intervention Group: 7%  Control Group: 14% | Intervention Group: 27.88 (9.24)  Control Group: 26.68 (7.32)  MIN= 18 and MAX=55 |
| Galante et al (2016) | UK and USA | General (English speakers) | Wales (57%) and White (89%), with North Americans (5%). | Females: 80% | Median= 34 years. MIN= 18, MAX= 79 |
| Halamova et al (2018) | Slovakia | General (Community) | N/A | Intervention Group:  Females: 85.7%, and Males: 14.2%  Control  Group:  Females: 85%, and Males: 15% | Intervention Group: 25.57 (11.76)  Control Group: 25.35 (6.32). |
| Drozd Filip et al (2014) | Norway | General participants | N/A | Males:  Intervention Group: (25%) Control Group: (24.5%).  Females:  Intervention Group: (75%) Control Group: (74.5%) | Intervention Group: 30.3 (7.2)  Control Group: 30.9 (9.1) |
| Koydemir et al (2016) | Turkey | General (Students) | Turkish background | Intervention Group:  Females: 40.9%  Males: 59%  Control Group:  Females: 44.4%  Males: 55.5% | 18.75 (1.03)  MIN= 17 and MAX= 23 |
| Sergeant and Mongrain (2014) | Asia, Canada, USA | General (Community) | Caucasian (44%) and Asian (35%). Most of the participants resided in Asia (37%), Canada (35%), or the United States (17%). | Females: 65% | 32.75 (12.23)  MIN= 20  MAX= 45 |
| Lappalainen et al (2023) | Finland | General (Lower Secondary School Students) | Finnish | Female:(66.7%), Male: (32.9%), Other/does not want to tell: (0.4%). | 15.01 (0.14)  MIN=14 MAX= 16 |
| Tay (2022) | Singapore | General (University Students) | Chinese: (89.08%) Malay: (1.72%) Indian: (8.05%) Others: (1.15%) | Males: (28.74%) Females: (71.26%) | 18-21 years: 71.84%  22-24 years: 28.16%  MIN = 18  MAX = 24 |
| Paetzold et al (2022) | Germany | Clinical (psychological distress and high at-risk mental state or treated episode of severe mental disorder) | White majority (German): 70%, White other: 9%, Mixed White Majority or White other (7%), Turkish 6%, Mixed other 4%, Middle East 2%, Asian 2%. | Females: (76%) Males: (24%) | 21.30 (2.84) Min = 14 Max = 25 |
| Qu et al (2022) | China | Clinical (children diagnosed with Autism) | 100% Chinese | Program Evaluation:  Males: 84.38%  Females: 15.63%  Focus group Interviews:  Males:83.33%  Females: 16.67% | Child Age (Years)  Program Evaluation: 3.16 (0.88)  Range = 1.68–5.16  Focus Group Interviews:  3.31 (0.93)  Range = 1.70–5.16 |
| Webb et al (2022) | USA | Undergraduate students | 44% White/European American, 37.3% Black/African American, 6.7% Hispanic or Latina, 5.3% multiethnic/racial, 4% Asian/Asian American, 1.3% American Indian/Alaska Native, and 1% identity not listed. | Females = 100% | 20.98 (2.38), MIN= 18 and MAX= 30 |
| Nawa and Yamagishi (2021) | Japan | General (Volunteers) | 100% Japanese | Female: 78% Male: 21.9% | 21.75 (1.48). MIN= 20 and MAX= 30 |
| Brouzos et al (2023) | Greece | General participants | 100% Greek | Females: 78%  Males: 21.9% | 33.07 (9.55)  Min = 20  Max = 65 |
| Pizarro-Ruiz et al (2021) | Spain | General (University Students) | 100% Spanish | Experimental Group:  Males:7.9%  Females: 92.1%  Control Group:  Males: 28%  Females: 72% | 22.06 (6.00)  MIN= 15 and MAX= 60 |
| Halamova et al (2020) | Slovakia | General participants | 100% Slovakians | Intervention Group:  Females: 84.6%  Males: 15.4%  Control Group:  Females: 85%  Males: 15% | Intervention Group: 26.23 (6.43)  Control Group: 25.35 (6.32) |
| Sampson et al (2020) | UK | General (University Students) | White (47%), Asian (25.8%), Mixed (11.3%), Black (3%) and Other (12.9%) | Male: (39%) Female: (61%) | 20.50 (2.21) Min = 18  Max = 30 |
| Greer et al (2019) | USA | Clinical (Cancer patients) | White/Caucasian: (82%) Hispanic or Latino: (7%) Black or African American: (7%) Asian or Pacific Islander: (2%) Prefer not to answer: (2%) | Male: 20%  Female: 80% | 25 (2.9). MIN= 18 and MAX= 29 |
| Tagalidou et al (2019) | Austria | General (University Students) | Austrian  Completers (24.5%)  Non-Completers (26.3%)  German  Completers (75.5%)  Non-Completers (68.4%) Other  Completers: --  Non-Completers: (Italian, Dutch, missing) (5.3%) | Females:  Completers: 85.16%  Non-Completers:  84.2%  Males:  Completers:14.2%  Non-Completers: 15.2% | 24.91 (8.22). MIN= 18 and MAX= 61 |
| Bronk et al (2019) | USA | General population | Caucasian:  Gratitude: 70.3%  Purpose: 81.0%  Control: 67.6%  Hispanic/Latino:  Gratitude: 9.5%  Purpose: 6.3%  Control: 8.5%  African American:  Gratitude: 9.5%  Purpose: 6.3  Control: 15.5%  Asian:  Gratitude: 6.8%  Purpose: 5.1%  Control: 4.2%  More than one ethnicity:  Gratitude: 4.1%  Purpose: 1.3%  Control: 4.2% | Male:  Gratitude: 43%  Purpose: 33% Control: 44%  Female:  Gratitude: 57% Purpose: 67% Control: 54% | Mean and SD of Gratitude Group: 26.27 (2.96)  Mean and SD of Purpose Group: 26.53 (3.01)  Mean and SD of Control Group: 25.42 (3.06)  MIN = 18  MAX = 30 |
| Gu et al (2022) | South Korea | General (Chinese International Students) | 100% Chinese | Males:12.5%  Females: 87.5% | M (SD): Compassion Focused Therapy (CFT)-based Intervention (CFI): 23 (2.45) Rational Emotive Behavior Therapy (REBT): 23.30 (2.36) Wait-list (WL): 22.25 (2.49)  CFI:  MIN = 19  MAX= 26 REBT:  MIN = 19  MAX = 27 WL:  MIN = 18  MAX = 28 |
| Alexiou et al (2021) | Greece | Clinical (Healthcare professionals with mild to very severe symptoms of depression, anxiety and stress) | 100% Greek | Females: Intervention (89.5%)  Control (89.5%) Men:  Intervention (11.5%)  Control (11.5%) | Intervention Group: 33.9 (8.69). Control Group: 31.7 (7.17). |
| Manicavasagar et al (2014) | Australia | General (young students) | 100% Australians | Females: 67.5% | 15.4 (1.7).  MIN= 12 and MAX= 18 |

**Multimedia Appendix 1: Basic Characteristics**
