# Supplementary material for: Application of Positive Psychology in Digital Interventions for Children, Adolescents, and Young Adults: Systematic Review and Meta-Analysis of Controlled Trials
Source: JMIR Ment Health. 2024 Aug 14;11:e56045. doi: 10.2196/56045 (PMC11358669; doi:10.2196/56045)
Supplement: Multimedia Appendix 2 [file mental_v11i1e56045_app2.docx]

| **Study** | **Title And Abstract** | **Introduction** | **Methods** | **Results** | **Discussion** | **Other Information** | **Total Score** | **Percentage** |
| --- | --- | --- | --- | --- | --- | --- | --- | --- |
| Mahalik et al (2022) | 1 | 2 | 4 | 2 | 3 | 1 | 13 | 38% |
| Krifa et al (2022) | 1 | 2 | 12 | 9 | 4 | 1 | 29 | 85% |
| Drabu et al (2022) | 1 | 2 | 5 | 3 | 1 | 0 | 12 | 35% |
| Lennard et al (2021) | 2 | 2 | 8 | 7 | 4 | 1 | 24 | 71% |
| Andersson (2021) | 2 | 2 | 10 | 5 | 4 | 1 | 24 | 71% |
| Beshai (2020) | 2 | 2 | 10 | 7 | 4 | 1 | 26 | 76% |
| Chilver and Gatt (2022) | 2 | 2 | 9 | 7 | 3 | 1 | 24 | 71% |
| Hussong et al (2020) | 2 | 2 | 10 | 6 | 4 | 1 | 25 | 74% |
| Halamova et al (2018) | 2 | 2 | 9 | 5 | 4 | 1 | 23 | 68% |
| Kelman et al (2018) | 2 | 2 | 8 | 7 | 4 | 1 | 24 | 71% |
| Hamm et al (2019) | 2 | 2 | 7 | 6 | 4 | 1 | 22 | 65% |
| Daugherty et al (2018) | 1 | 2 | 9 | 6 | 4 | 1 | 23 | 68% |
| Halamova et al (2020) | 2 | 2 | 8 | 5 | 4 | 1 | 22 | 65% |
| Kappen et al (2019) | 1 | 2 | 10 | 6 | 4 | 1 | 24 | 71% |
| Galante et al (2016) | 2 | 2 | 8 | 5 | 4 | 1 | 22 | 65% |
| Halamova et al (2018) | 1 | 2 | 5 | 6 | 4 | 1 | 19 | 56% |
| Drozd Filip et al (2014) | 2 | 2 | 9 | 7 | 3 | 1 | 24 | 71% |
| Koydemir et al (2016) | 1 | 2 | 9 | 3 | 3 | 1 | 19 | 56% |
| Sergeant and Mongrain (2014) | 1 | 2 | 9 | 7 | 3 | 1 | 23 | 68% |
| Lappalainen et al (2023) | 2 | 2 | 10 | 8 | 4 | 1 | 27 | 79% |
| Tay (2022) | 2 | 1 | 10 | 7 | 3 | 1 | 24 | 71% |
| Paetzold et al (2022) | 1 | 2 | 13 | 6 | 4 | 2 | 28 | 82% |
| Qu et al (2022) | 1 | 2 | 9 | 6 | 2 | 1 | 21 | 62% |
| Webb et al (2022) | 0 | 2 | 12 | 8 | 2 | 1 | 25 | 74% |
| Nawa and Yamagishi (2021) | 1 | 2 | 12 | 4 | 3 | 1 | 23 | 68% |
| Brouzos et al (2023) | 0 | 2 | 9 | 4 | 3 | 0 | 18 | 53% |
| Pizarro-Ruiz et al (2021) | 0 | 2 | 7 | 5 | 3 | 2 | 19 | 56% |
| Halamova et al (2020) | 1 | 2 | 8 | 7 | 3 | 1 | 22 | 65% |
| Sampson et al (2020) | 2 | 2 | 13 | 7 | 3 | 2 | 29 | 85% |
| Greer et al (2019) | 2 | 2 | 9 | 3 | 3 | 0 | 19 | 56% |
| Tagalidou et al (2019) | 1 | 2 | 10 | 7 | 2 | 2 | 24 | 71% |
| Bronk et al (2019) | 0 | 2 | 6 | 5 | 2 | 0 | 15 | 44% |
| Gu et al (2022) | 1 | 2 | 8 | 7 | 2 | 1 | 21 | 62% |
| Alexiou et al (2021) | 0 | 2 | 7 | 6 | 3 | 0 | 18 | 53% |
| Manicavasagar et al (2014) | 2 | 2 | 6 | 6 | 3 | 0 | 19 | 56% |

**Multimedia Appendix 2: Quality Assessment**
