# Supplementary material for: Application of Positive Psychology in Digital Interventions for Children, Adolescents, and Young Adults: Systematic Review and Meta-Analysis of Controlled Trials
Source: JMIR Ment Health. 2024 Aug 14;11:e56045. doi: 10.2196/56045 (PMC11358669; doi:10.2196/56045)
Supplement: Multimedia Appendix 3 [file mental_v11i1e56045_app3.docx]

| **Study** | **Reference Theory** | **Intervention (Main Focus)** | **Intervention (Type of Activity)** | **Setting** |
| --- | --- | --- | --- | --- |
| Mahalik et al (2022) | N/A | Increase fathers’ sense of purpose | On the Fatherhood Project's webpage, participants are presented with an opportunity for reflection and self-discovery. They were first asked to engage with "10 Facts about Father Engagement" and then encouraged to express their thoughts on father involvement without constraints on length or a right/wrong framework.  The second part of this psychoeducation exercise seek to deepen fathers' understanding of the impact they can have on their children's lives. By prompting fathers to consider their long-term goals and aspirations for their children, recent positive experiences, core values, character strengths, and areas for skill development, the exercise fostered introspection to encouraged fathers to explore the purpose of their roles in their children's lives, emphasizing the multifaceted nature of fatherhood. | Online/ Webpage. |
| Krifa et al (2022) | N/A | Assess the efficacy of an internet-based positive psychology program in improving mental health, including stress, anxiety, and depression, as well as overall well-being, among Tunisian university students during the COVID-19 pandemic, with the aim of bolstering the mental health of this at-risk group. | The CARE (Coherence, Attention, Relationship, and Engagement) multi-component positive psychology self-help program was structured around three key processes aimed at enhancing well-being. These processes included the redirection of focus toward the positive and fulfilling aspects of life, fostering compassion and self-compassion, and engaging in meaningful actions. Expert videos were included to provide insights and perspectives, enhancing the educational aspect. Psychoeducation helped participants understand the psychological principles underpinning the practices. The core of the program lied in positive psychology exercises and one to two weekly activities assigned to the students. The entire program was eight sessions, with each session requiring about 45 minutes of commitment per week to complete the online activities. These sessions consisted of a blend of expert insights and hands-on exercises. Importantly, if participants hadn't visited the program's website on their own initiative, they were prompted with email reminders to encourage them to log in and engage with the activities throughout the week. | Online |
| Drabu et al (2022) | N/A | Effectiveness of a concise online self-compassion training program in reducing both overt and underlying self-criticism, enhancing pain tolerance, and decreasing the tendency to participate in self-harming behaviors among individuals who engage in or contemplate non-suicidal self-injury (NSSI). | In the training group, participants were initially provided with a concise introduction to the mindful self-compassion practice. Following this, they engaged in a single self-compassion-based guided meditation session known as "Loving Kindness for Ourselves," which is part of the Mindful Self-Compassion (MSC) program. This guided meditation session had a duration of 20 minutes. Subsequently, participants were instructed to spend the next 5 minutes crafting a self-compassionate letter to themselves. All participants underwent a distress protocol, and their next lab session was scheduled for one week later, where they completed the T2 measurements. The first lab session spanned 90 minutes, while the second session lasted for 20 minutes. During the interim between the two training sessions, the training group received daily email links that included audio-guided meditations for them to follow, accompanied by the task of writing a daily self-compassionate letter. These emails also solicited feedback and experiences related to their daily practice. In the initial lab session, participants experienced the same guided meditation on the 2nd and 3rd days as they did during the lab session. For the 4th and 5th days, they received the Compassionate Body Scan meditation. Finally, on the 6th and 7th days, they were provided with the Affectionate Breathing meditation. | Online |
| Lennard et al (2021) | Acceptance and commitment theory | Assess the efficacy of online materials aimed at enhancing self-compassion in mothers with infants (< 2 years). | The intervention provided online self-compassion resources, including two instructional videos and a downloadable tip-sheet. The first video offered psychoeducation on self-compassion in the context of motherhood, while the second video featured a guided self-compassion visualization exercise. The tip-sheet contained practical strategies for nurturing self-compassion, such as empathizing with others, being receptive to giving and receiving compassion, envisioning support from a compassionate person, recognizing shared experiences, and scheduling self-kindness activities. Participants in the intervention group had unrestricted access to these materials during the study, with seven weekly SMS reminders to encourage their utilization, thereby supporting mothers in enhancing self-compassion. | Online/ SMS/ Videos |
| Andersson (2021) | Affect theory, Attachment theory | Assess the impact of a smartphone app featuring a compassion-focused psychological program on self-compassion, stress levels, emotional awareness, and overall distress in stressed university students. | The Compassion Mindset Intervention is a comprehensive program designed to cultivate compassion, emotional awareness, emotion regulation, and resilience to stress. It was six weekly modules, each focused on a specific theme: (1) understanding compassion, (2) Gilbert's three-circle model for emotion regulation, (3) self-compassion, (4) nurturing a compassionate mindset, (5) extending compassion to others, and (6) practicing gratitude and wisdom. Each module comprises five core elements: theoretical insights, guided meditations, reflective exercises, breathing practices, and practical exercises that promote the integration of a compassionate approach into daily life. Participants were encouraged to allocate 10-15 minutes per day to the program over six weeks. The Mindfulness App program, on the other hand, consisted of a 7-day introduction to mindfulness, followed by a self-guided practice spanning five weeks with the aid of six guided meditations, breathing exercises, and body scans. Additionally, participants were offered 15 supplementary exercises for added variety. The mindfulness training was self-administered, and participants were encouraged to invest 10-15 minutes daily for a total of six weeks in this practice. These programs aimed to enhance the participants' well-being and emotional resilience through structured and accessible training modules. | Smartphone-based (App) |
| Beshai (2020) | N/A | Evaluated the effectiveness of a new program, Mind-OP, combining mindfulness and self-compassion approaches. | Each weekly module comprised of psychoeducational videos introducing new concepts, followed by audio guided meditations aimed at instilling these concepts. Each module included a scheduler for setting meditation schedules, as well as motivational interviewing exercises to boost commitment to the practice. Week 1 included two 5-minute videos and one 5-minute guided meditation. Week 2 centered on mindfulness of body and thoughts, incorporating video and audio components. Week 3 involved two videos and two guided meditations. In Week 4, one video discussed self-kindness and self-pity, and another addressed self-kindness in the context of stress. | Internet-based |
| Chilver and Gatt (2022) | N/A | Implement online Multi-component Positive Psychological Intervention (MPPI) within a university sample. Examined whether the initial levels of resiliency, denoting the support systems accessible to participants, would influence the intervention's impact. | The intervention comprised three modules, each spanning two weeks with distinct activities. Firstly, the self-compassion module guided participants to reflect on past experiences causing self-doubt, fostering self-compassion by writing a compassionate letter to themselves. Secondly, participants brainstormed and performed acts of kindness for others. Thirdly, they recalled significant life events, conveying them as if to a friend. In the control group, the self-esteem module focused on finding positivity in negative situations. The acts of novelty module encouraged changes in routine. The neutral reminiscence module used prompts related to personal strengths and emotional moments rather than overcoming challenges. | Online |
| Hussong et al (2020) | Eisenberg’s theory of parent emotion socialization | Assess the effects of the Gratitude Conversations program on intended mediators (parental intentions, confidence, and knowledge), as well as specific outcomes (utilization of gratitude strategies, parenting behaviors, and children's gratitude experiences). | The Gratitude Conversations program served as an online parenting training tool rooted in cognitive-behavioral change principles. It encompassed multiple components within a 45-minute framework, designed to engage participating parents effectively. The program commenced with psychoeducation, emphasizing the significance of gratitude, its core elements, challenges children face in expressing gratitude, and the CARE and SHARE strategies for parent-adolescent communication. Moreover, the program featured videos of three parent-youth dyads, illustrating both poor and exemplary gratitude conversations and missed opportunities. After viewing, parents engaged in reflection, identifying aspects they found favorable or unfavorable within these conversations. The dyads' interactions were critiqued with respect to the application of CARE and SHARE strategies. Parents did set specific change goals, select 1-2 strategies for implementation, address potential obstacles, and partake in program evaluation. | Online |
| Halamova et al (2018) | Emotion-focused therapy theory | Assess the short-term and enduring effects of an internet-based adaptation of Emotion Focused Training for Self-Compassion and Self-Protection (EFT-SCP) on self-compassion, self-criticism, and their respective components within a non-clinical sample. | EFT-SCP integrated elements from Compassion Mind Training (CMT) and the MSC with principles from Emotion-focused therapy. CMT involved 6 to 12 weekly sessions lasting 1-2 hours, with group meetings held once or twice weekly. MSC comprised 2-2.5-hour group sessions weekly for eight weeks, with daily home exercises. In Emotion-focused therapy, therapists guided clients to connect with primary adaptive emotions, allowing them to access valuable information and action tendencies to facilitate problem-solving within the therapeutic process. | Online |
| Kelman et al (2018) | N/A | Investigate potential distinctions in various outcomes between Cognitive-Behavioral Therapy (CBT) and CMT among women who are either perinatal or planning to become pregnant. | The online materials consisted of interactive lessons addressing four key topics: (a) thoughts, (b) activities, (c) assertiveness, and (d) sleep. Participants received these materials in subsequent emails over the 2-week course to reinforce the concepts covered in the instructional part of the course. | Online |
| Hamm et al (2019) | Motivational theory of life-span development (Heckhausen’s theory) | A goal engagement intervention aimed at improving self-regulatory SSC (Selective Secondary Control) processes to a group of students with different levels of high school grades (ranging from low to high) and optimism (varying from low to high). | The goal engagement treatment comprised a one-hour session with three stages. First, the activation phase prompted reflection on academic successes and failures to enhance the treatment's relevance. Then, the induction phase involved a video presentation on self-regulatory SSC strategies (anticipation, prioritization, and persistence). Lastly, the consolidation stage involved a writing activity where participants set academic goals, wrote about anticipated positive emotions, why their goals were a priority, and a personal persistence model. | Online |
| Daugherty et al (2018) | N/A | Perform a feasibility assessment of a smartphone EMI aimed at nurturing hope to enhance overall well-being. Investigate the effectiveness of a hope EMI delivered through a versatile mobile app system in elevating hope levels and improving heuristic and eudemonic well-being, including emotional and psychological well-being. | Participants in the intervention group received a 28-day mobile app intervention that provided random in-the-moment hope notifications, which included hope picture-statements, hope statements, and questions from the Snyder Hope scale. Additionally, they had access to peer stories of hope obtained from university students and selected with input from the research team. | Smartphone app based. |
| Halamova et al (2020) | N/A | Assess the short-term and lasting effects of an online intervention rooted in the MSC program on self-compassion, self-criticism, and self-reassurance in individuals without clinical conditions. | The program consisted of both in-class and home-based exercises, including formal practices like loving-kindness meditation and informal practices like self-compassionate gestures when distressed. Participants met weekly for 2-2.5 hours over eight weeks, with the option to attend a half-day silent meditation retreat. The program primarily focused on teaching self-compassion skills, dedicating only one session to mindfulness. Those in the MSC group were prompted via email to complete daily exercises for 14 consecutive days, each taking at least 15 minutes. These exercises were accessible on any device through an email link, featuring topics like "How Would You Treat a Friend?" and "Appreciating Yourself" on the final day. | Online |
| Kappen et al (2019) | N/A | Whether engaging in mindfulness practices might lead to subsequent impacts on the dynamics of a romantic relationship. | The mindfulness intervention included psychoeducation, daily reporting of negative relationship incidents, and daily mindfulness exercises. Participants practiced mindfulness every day, regardless of relationship incidents. The mindfulness exercises focused on present awareness, breathing, and bodily sensations. Guided audio instructions were provided for 8 to 10 minutes. Participants were encouraged to apply mindfulness to daily activities. | Online |
| Galante et al (2016) | Broaden and Build theory; Empathy-Altruism theory | Assessing the Efficacy of an Online Loving-Kindness Meditation (LKM) Training Program for Enhancing Well-Being. | Participants engaged in real-time practice by mirroring the exercises demonstrated by actors in video sessions. Clear voiceover instructions were provided, detailed enough to allow participants to follow along with their eyes closed if they preferred. New 10-minute video sessions were made accessible each weekday, totaling 20 sessions in the course. Automated email reminders were sent to participants who fell behind their session schedule, although they had the flexibility to catch up. Previous sessions were also available for review, and written summaries in PDF format were provided. LKM exercises primarily involved repetitive phrases (e.g., "May you be well and happy") or visualizations of radiating light from oneself to others, aimed at cultivating feelings of loving-kindness toward the meditation's focal point. | Online/ Internet Based |
| Halamova et al (2018) | N/A | Assess the short-term and long-term effects of an internet-based Mindfulness-Based Intervention (MBI) involving Mindfulness-Based Stress Reduction (MBSR) practices on self-compassion, self-criticism, and self-reassurance in a non-clinical sample. | The MBSR program encompassed formal mindfulness practices, including body scan, yoga, sitting meditation, and loving kindness meditation, alongside informal mindfulness practices integrated into daily activities like washing dishes and driving. Structured group sessions provide training in both formal and informal mindfulness practices, allowing participants to share their experiences with mindfulness in daily life. The MBSR program spans eight weeks, involving weekly 2.5-hour sessions and a one-day six-hour session. Most exercises were audio-guided, while yoga exercises are presented visually. All materials, including audio recordings, are available in Slovak. Participants received the intervention through an accessible link via email, with a sequence starting with "Body Scan," followed by "Sitting meditation," "Yoga," and concluding with "Loving Kindness Meditation" and "Informal mindfulness practices." Participants in the MBI group practiced daily for 15 consecutive days, dedicating 15 minutes to each exercise. | Online/ Internet Based |
| Drozd Filip et al (2014) | N/A | Assess the impacts of a web-based positive psychology program focusing on gratitude, enjoyable activities, personal strengths, achievement, acts of kindness, optimism, flow, attributions, and mindfulness. | 'Better Days' (BD) is a fully automated internet-based program aimed at enhancing daily life and well-being, drawing from principles of positive psychology. The program is structured into 13 sessions spanning four weeks, each designed to be completed in approximately 10 minutes, considering typical website visit durations. These concise 10-minute sessions were crafted to effectively deliver positive psychology interventions (PPIs) while ensuring participant engagement. Additionally, participants received homework assignments to facilitate the integration of intervention materials into their daily routines, promoting sustainable improvements. Each session was divided into psychoeducational and exercise sections, offering a variety of interventions, totaling nine diverse strategies such as gratitude, acts of kindness, optimism, coping techniques, living in the present moment, and character strengths. | Internet-based |
| Koydemir et al (2016) | Broaden-and-build theory of emotions. Theory of flow. Seligman’s well-being theory (2011). | Investigated the impact of an 8-week online intervention that focuses on individual strengths in enhancing the subjective and psychological well-being of first-year university students. | The intervention program encompassed five modules distributed over an 8-week period, covering the following topics: 1. Identifying strengths and setting goals. 2. Enhancing emotion regulation and boosting positive emotions. 3. Cultivating social support, improving communication, and nurturing positive relationships. 4. Developing effective problem-solving skills and decision-making abilities. 5. Fostering motivation, experiencing flow, and practicing gratitude. These modules were delivered through a diverse range of methods, including psychoeducational webinars, audio and text materials, hands-on experiential activities, game-based exercises, instructional videos, and interactive activities involving personal sharing. | Online/Webinars/Videos |
| Sergeant and Mongrain (2014) | N/A | Compared the impact of an empirically based online optimism exercise to a neutral diary-writing exercise on psychological well-being. It investigated whether enhancing optimism, a potentially malleable trait related to well-being, could have lasting positive effects. The study also considered trait pessimism as a potential moderator of these effects. | The experimental condition included two alternating exercises aimed at cultivating optimism: (a) encouraging a cognitive bias toward recalling positive life experiences, and (b) fostering a perspective that deems goals achievable and valuable. In the first exercise, participants were instructed to list five things that brought joy and enrichment to their lives, along with three ways to find positivity in challenging situations. The next exercise, which occurred 48 hours later, involved briefly describing a goal to be achieved within the next day or two and outlining the necessary steps. Participants continued to alternate between these exercises throughout the 3-week intervention period. | Online |
| Lappalainen et al (2023) | N/A | The primary goal of this study was to assess the impact of the online HOPE intervention on help-seeking attitudes and intentions among young adults (aged 18 to 24) at a Singaporean university. Additionally, the study conducted a process evaluation of the online HOPE intervention. | Two intervention groups were tested: the iACT student coach + virtual coach group, which received a five-week Youth Compass online program with two 45-minute video calls from a student coach and support from a virtual coach (chatbot and SMS coaching), and the iACT virtual coach group, which received the same program with one 15-minute video call from a student coach and support from the virtual coach. The student coaches, who were psychology students trained in ACT, coached around eight randomly selected adolescents, while the virtual coach provided SMS support and guidance throughout the program. | Online |
| Tay (2022) | Bandura's Self-Efficacy Theory | The primary goal of this study was to assess the impact of the online HOPE intervention on help-seeking attitudes and intentions among young adults (aged 18 to 24) at a Singaporean university. Additionally, the study conducted a process evaluation of the online HOPE intervention. | The HOPE intervention was a web-based platform comprising four sessions, with participants encouraged to complete two sessions weekly. Each session included quizzes, videos, and information related to mental health. The first session provided insights into depression, covering myths, causes, symptoms, self-help strategies, and treatments. The second session introduced strength-based, affect-based, and gratitude exercises from established psychology manuals. The third session focused on anxiety disorders, discussing myths, causes, symptoms, self-help strategies, and treatments. The final session addressed relaxation techniques and cognitive management. Weekly reminders via WhatsApp and email were sent to participants to promote intervention compliance. | Online/ Website |
| Paetzold et al (2022) | Attachment theory, and social mentality theory | Evaluate a compassion-focused intervention for improving resilience in young help-seeking individuals through a combination of online and in-person sessions and investigate whether participants' initial characteristics are linked to the intervention's mechanisms and outcomes. | Participants engaged in 4 individual smartphone app-based sessions with daily training. The intervention offered two tracks, basic and elaborate, depending on initial impressions and experiences. The basic track aimed at creating calmness and safety through breathing exercises and soothing imagery, while the elaborate track extended these practices to include self-compassionate imagery and writing. The intervention included 3 guided sessions introducing compassion-focused principles, practical tasks, and feedback on progress. Sessions were conducted in person or via video calls, with in-person sessions held in dedicated rooms, and home-based sessions for video calls. | Hybrid (EMI and guided face-to-face sessions) |
| Qu et al (2022) | N/A | Assess Chinese parents' views on their engagement in a culturally tailored group-based parent coaching program delivered through telehealth. This evaluation aims to gauge parents' levels of satisfaction, acceptability, appropriateness, and the feasibility of the intervention. | The Group-based Parent Coaching Intervention is a culturally adapted 12-week program structured around Parent-mediated Early Start Denver Model (P-ESDM) and delivered in Chinese through the Canvas online platform. Each module covers diverse topics related to autism and child development. To cater to Chinese parents' needs, the program utilizes a family capacity-building approach, incorporating four intervention components: asynchronous lectures with practice manuals, demonstration and commentary videos, web-based Q&A sessions, and optional homework. These resources enhance parents' understanding and application of strategies for children with Autism Spectrum Disease (ASD). The active comparison group navigated the program at their own pace, while the treatment group (web+group therapy) followed the same program and engaged in weekly 1.5-hour virtual group therapy sessions via DingTalk, guided by a standardized protocol. | Telehealth. Canvas Online Management System. |
| Webb et al (2022) | Embodiment theory | Assess the workability, approval, and initial effectiveness within the group of ethnically diverse college women with higher body weight who successfully completed the integrative online mind-body program (i.e., per-protocol analysis). | Participants in the experimental group were instructed to engage in yoga practice at least three times a week, following 15-minute Curvy Yoga Studio video links sent via email. Week 1 focused on a "Morning Wake-up" practice, incorporating gentle yet energizing yoga sequences. Week 2's "Find Your Steady and Sweet" practice emphasized balance and lovingkindness. Week 3's "Living Your Body Positive Intention" added new poses and affirmations. In week 4, participants could choose any of the previous videos. They were encouraged to set intentions, use props, and practice at their own pace. Participants also kept a daily body gratitude journal. | Online |
| Nawa and Yamagishi (2021) | Broaden-and-build theory. Self-determination theory. | Examined the effects of online gratitude journal intervention on the academic motivation of university students. | Participants were required to access the online system daily for two weeks. After logging in, a calendar displayed daily tasks. By clicking on a task (e.g., Diary), participants entered information on task-specific pages. They had flexibility in terms of location, time, duration, and device for access, like laptops or smartphones. For the first 6 days of each week, the gratitude group described up to 5 events or thoughts making them feel grateful. They also rated various aspects of their daily lives, such as stress and happiness, using sliding bars. Control group participants performed similar self-assessments but were not tasked with the gratitude journal. An instructional video on system usage was provided and encouraged before starting the activities. | Online/ Web-based |
| Brouzos et al (2023) | N/A | Assessed the efficacy of an online group positive psychology intervention (OPPI) in alleviating the psychological consequences of the COVID-19 pandemic and its associated containment measures. | The "Staying Home – Feeling Positive" program discussed in this paper is a two-week online group intervention consisting of six sessions. It draws from the positive psychology framework while also integrating elements from cognitive-behavioral therapy. The intervention is conducted through online teleconferencing software (e.g., Skype) with small groups of 5–7 participants. Each session has a duration of approximately 50 minutes, and sessions are held three times a week. Each session focuses on a distinct topic, such as self-protection, relaxation techniques, mindfulness coping, fostering a positive mindset, promoting empathy, altruism, and love, and concluding the program. | Online |
| Pizarro-Ruiz et al (2021) | Theory of mindfulness | Assess the impact of a brief Mindfulness-Based Intervention (MBI) administered via a smartphone app on various factors associated with positive job performance, including traits of mindfulness, emotions, forgiveness, character strengths, and life satisfaction. | The experimental group was directed to install and utilize a readily available smartphone app called "Aire Fresco," which offered guided mindfulness sessions. They were instructed to engage in one session each day for the following 14 days. These mindfulness sessions typically lasted around 15 minutes and 47 seconds, with the longest session being 26 minutes and 8 seconds, and the shortest lasting 11 minutes and 47 seconds. In contrast, the control group received similar instructions but were required to download a different smartphone app, "Lumosity." This app provides a variety of activities collectively referred to as "mind training," encompassing games targeting attention, memory, speed, flexibility, and problem-solving. Participants in the control group were asked to dedicate approximately 16 minutes per day to using this app during the same 14-day period as the experimental group. | Smartphone-app (Aire Fresco (Fresh Air);  (Lumosity). |
| Halamova et al (2020) | N/A | Assess the effects of a condensed online version of CMT on self-compassion and self-criticism within a non-clinical sample. | Participants in the online intervention were tasked with completing a daily CMT exercise over the course of 13 consecutive days, starting the day after they were assigned to the intervention group. Each participant received an email instructing them to access the CMT exercise through a provided link. The exercises were structured with the aim of enhancing their potential impact on well-being. After each exercise, participants were prompted with free-text questions encouraging them to reflect on how they could integrate the exercise into their daily lives and describe certain aspects of their experience, such as their "safe place." Additionally, these post-exercise tasks served as a fidelity check, sending email reminders to those who hadn't completed the exercise. The exercises were selected from a variety of options available in Compassionate Mind Training and presented in a specific order, including "Soothing Rhythm Breathing," "Compassionate Body Scan," "Imaging the Self-Critical part of Self," "Creating a Safe Place," "Compassionate Colour," "Developing the Inner Compassionate Self," "Working with Troubled Self," "Working with Anxious Self," "Compassion Flowing into you from Others," "Compassion Flowing Out," "Compassionate Letter Writing," "Compassionate Dialogue Writing," and "Creating your Ideal Caring-compassionate Image." | Online |
| Sampson et al (2020) | Festinger’s Social Comparison Theory | Assess the impact of visual-focused social media on body and facial dissatisfaction and explore the relationship between increased social media use and these dissatisfactions. | Two image sets were created: the experimental group had 60 attractive smiles from Instagram users (rated for attractiveness, appeal, quality, and enjoyment), and the control group had 60 nature images. Participants viewed these images on Apple iPads through the Instagram app. | Social networking Site (Instagram Users) |
| Greer et al (2019) | Stress and Coping theory and the Broaden-and-Build theory | Assess the interaction and user-friendliness of the Vivibot chatbot and examine the initial impact of positive psychology skills provided by Vivibot on important psychosocial well-being factors in young adults undergoing cancer treatment. | Vivibot is an automated chatbot delivering a prewritten cognitive and behavioral intervention to enhance positive emotions, based on Moskowitz et al.'s work. Users were informed they were interacting with a computer system and repeatedly reminded of this. The intervention covers eight positive psychological skills, adapted into seven conversational teaching lessons and seven practice lessons, each repeated three times over 28 days. The control group received delayed access to the full Vivibot content, with a message notifying them of a 4-week delay. | Vivibot chatbot and Facebook Messenger |
| Tagalidou et al (2019) | Humor theory.  Wellenzohn's solving stressful situations in a humorous way. | Evaluate the relative effectiveness of both humor-based interventions compared to the placebo control condition.  Investigate the parity in efficacy between the two humor-based interventions and the three good things intervention.  Assess whether the three good things intervention surpasses the placebo control condition. | "Three Funny Things" involves documenting three daily humorous incidents and related emotions for a week. "Coping Humor" focuses on humorously resolving daily stressors through guided techniques for seven days. "Three Good Things" requires recording three positive daily experiences. | Web-based |
| Bronk et al (2019) | Broaden-and-build theory | Assess the effectiveness of two web-based toolkits (Purpose and Gratitude) aimed at nurturing a sense of purpose in young adults. | The Purpose Toolkit had three activities, each lasting 15-20 minutes over three days. Participants viewed videos, reflected on their values and future goals, and imagined their best possible future life, even designing a symbolic tattoo.  The Gratitude Toolkit, also lasting three days for 15-20 minutes per day, included a gratitude walk, listing things they were grateful for and why, learning about benefit appraisals, and writing a letter of gratitude to someone who had helped them. | Online |
| Gu et al (2022) | Attachment theory, and social mentality theory | Examine the impact of an online Compassion focused therapy (CFT)-based intervention (CFI) and rational emotive behavior therapy (REBT) on depression, anxiety, and shame using a Randomized Controlled Trial (RCT) with Chinese international students who exhibit elevated levels of self-criticism. | The CFI group engaged in a 4-week program involving weekly 2-hour online individual counseling sessions. This program, primarily rooted in CFT, focused on cognitive education related to the three emotion regulation systems, identifying and validating the inner critical voice, as well as practicing imagery and meditation. These meditation practices included mindfulness and compassion meditation, drawn from the Mindful Self-Compassion program. All participants were required to dedicate 20 minutes daily to home practice (guided meditation provided as MP3 files). During the 2-week follow-up, participants were encouraged to maintain their home practice.  The REBT group also took part in a 4-week program based on self-help approach involved attending a 2-hour online lecture each week. During these lectures, a sample case and case analysis were presented, and participants were tasked with applying these principles to their own lives as home practice. | Online |
| Alexiou et al (2021) | N/A | Assess the efficacy of a 21-day online positive psychology program for Greek healthcare professionals experiencing depression, anxiety, stress, or burnout. | Week one involved a "Three Good Things" exercise where participants noted three positive occurrences each day along with a causal explanation. Week two featured "Acts of Kindness," requiring participants to perform five kind acts on a single day. In the third week, participants engaged in a "Best Possible Self" exercise, envisioning their ideal future selves across various life domains and outlining steps to actualize these visions. | Online (Website) |
| Manicavasagar et al (2014) | N/A | Examine the practicality of introducing the Bite Back program, a multifaceted positive psychology initiative created by the Black Dog Institute, as a well-being intervention for youth. | Bite Back is an online platform designed for teenagers, offering a blend of engaging activities and educational content spanning nine positive psychology areas: gratitude, optimism, flow, meaning, hope, mindfulness, character strengths, healthy living, and positive relationships. Additionally, the platform supplies information on the advantages of enhancing well-being, strategies to cultivate proficiencies in each positive psychology domain, connects users to additional pertinent resources, and facilitates discussions through comments and online forums. Geared towards adolescents aged 13 to 17, the platform maintains a pre-moderation process, ensuring that all comments and uploads are reviewed and approved before being visible to the public. | Online |

### Multimedia Appendix 3: Characteristics of the Positive Psychology Intervention
