# Supplementary figures and images for: Application of Positive Psychology in Digital Interventions for Children, Adolescents, and Young Adults: Systematic Review and Meta-Analysis of Controlled Trials
Source: JMIR Ment Health. 2024 Aug 14;11:e56045. doi: 10.2196/56045 (PMC11358669; doi:10.2196/56045)

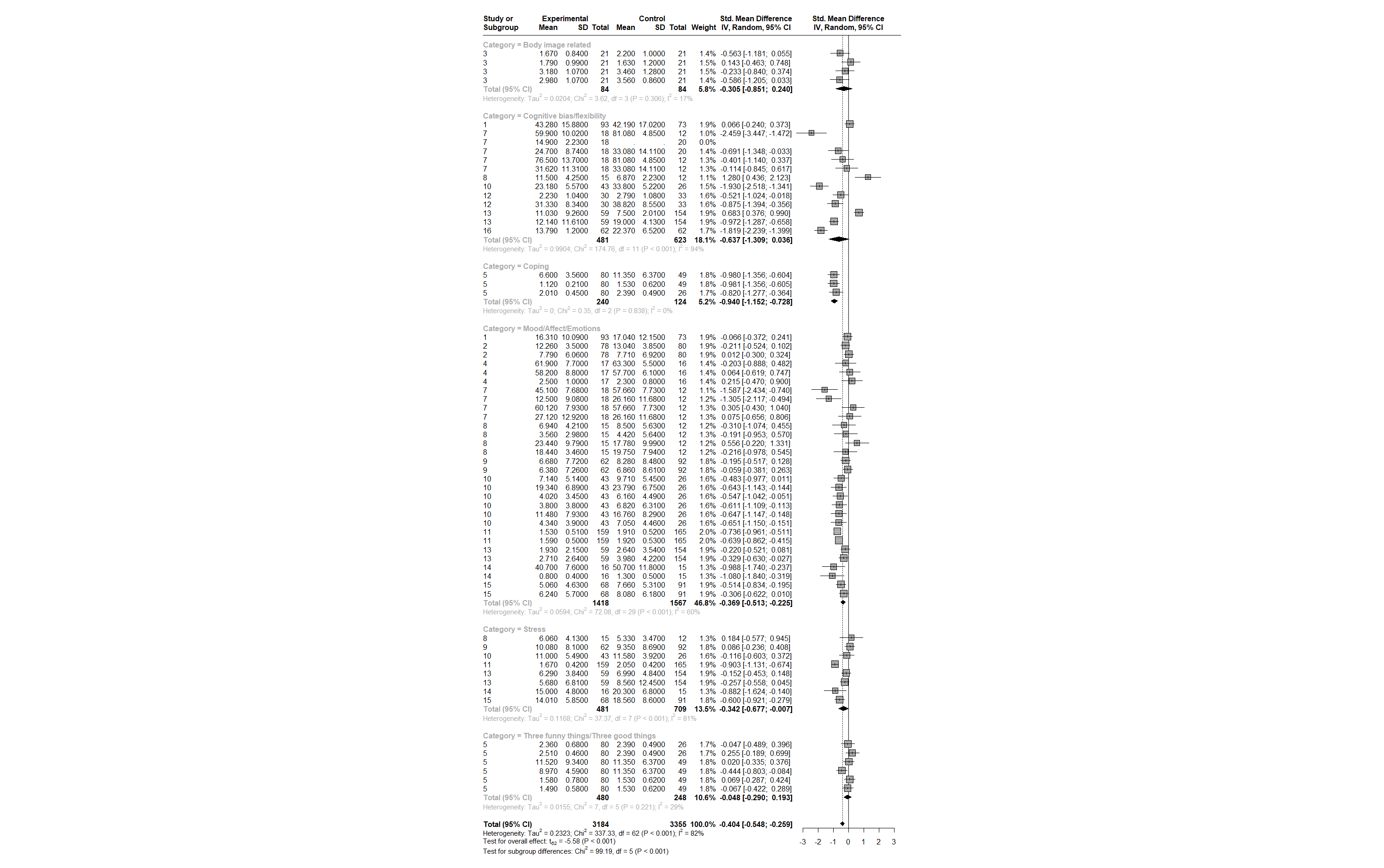

Supplement: Multimedia Appendix 4 [file mental_v11i1e56045_app4.png]

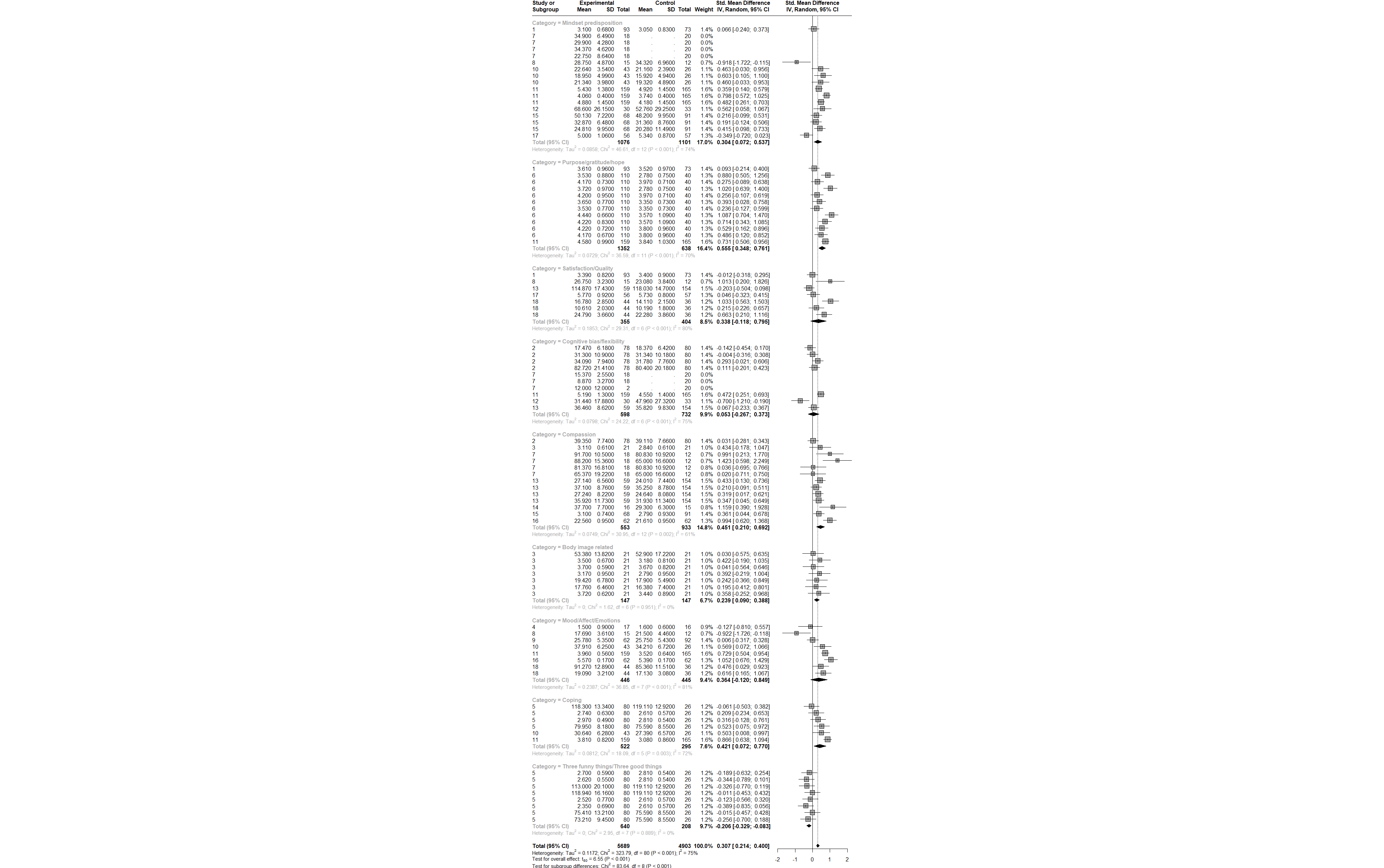

Supplement: Multimedia Appendix 5 [file mental_v11i1e56045_app5.png]
